# Association of Antibacterial Susceptibility Profile with the Prevalence of Genes Encoding Efflux Proteins in the Bangladeshi Clinical Isolates of *Staphylococcus aureus*

**DOI:** 10.3390/antibiotics12020305

**Published:** 2023-02-02

**Authors:** Tanjina Akter Suma, Nushrat Alam, Sheikh Zahir Raihan, Md. Al Zahid, Shankar Chandra Mandal, Fahrin Jahan Suchana, Ripa Kundu, Anwar Hossain, Md. Abdul Muhit

**Affiliations:** 1 Department of Clinical Pharmacy and Pharmacology, Faculty of Pharmacy, University of Dhaka, Dhaka 1000, Bangladesh; 2 Department of Fisheries, University of Dhaka, Dhaka 1000, Bangladesh

**Keywords:** *Staphylococcus aureus*, MRSA, efflux pump genes, antibiotic resistance, MDR *S. aureus*

## Abstract

Expelling antibiotic molecules out of the cell wall through multiple efflux pumps is one of the potential mechanisms of developing resistance against a wide number of antibiotics in *Staphylococcus aureus*. The aim of this study was to investigate the association between the antibiotic susceptibility profile and the prevalence of different efflux pump genes i.e., *norA, norB, norC, mepA, sepA, mdeA, qacA/B*, and *smr* in the clinical isolates of *S. aureus*. Sixty clinical isolates were collected from a tertiary level hospital in Bangladesh. The disc diffusion method using ten antibiotics of different classes was used to discern the susceptibility profile. polymerase chain reaction (PCR) was employed to observe the resistance patterns and to detect the presence of plasmid and chromosomal encoded genes. Among the clinical isolates, 60% (36 out of 60) of the samples were Methicillin-resistant *Staphylococcus aureus* (MRSA), whereas 55% (33 out of 60) of the bacterial samples were found to be multi-drug resistant. The bacteria showed higher resistance to vancomycin (73.33%), followed by ciprofloxacin (60%), cefixime (53.33%), azithromycin (43.33%), and amoxicillin (31.67%). The prevalence of the chromosomally-encoded efflux genes *norA* (91.67%), *norB* (90%), *norC* (93.33%), m*epA* (93.33%), s*epA* (98.33%), and *mdeA* (93.33%) were extremely high with a minor portion of them carrying the plasmid-encoded genes *qacA/B* (20%) and *smr* (8.33%). Several genetic combinations of efflux pump genes were revealed, among which *norA + norB + norC + mepA + sepA + mdeA* was the most widely distributed combination among MRSA and MSSA bacteria that conferred resistance against ciprofloxacin and probably vancomycin. Based on the present study, it is evident that the presence of multiple efflux genes potentiated the drug extrusion activity and may play a pivotal role in the development of multidrug resistance in *S. aureus*.

## 1. Introduction

Antibiotic resistance is a major public health concern in the treatment of *Staphylococcal* infections, particularly the infections caused by methicillin-resistant *S. aureus* (MRSA). The widespread indiscriminate use of antimicrobial agents combined with the transmission of a significant portion of the organism through person-to-person contact generally develops antimicrobial resistance in the clinical isolates [1,2].

The most current *S. aureus* epidemiology is focused on the rise and dissemination of MRSA in clinical settings and in the population [3]. However, methicillin-susceptible *S. aureus* (MSSA) has been a major cause of outbreaks and the global expansion in healthcare settings over the last century, and it is still one of the most common bacteria found in hospital infections. A recent point-prevalence survey conducted in acute care hospitals in 33 countries and coordinated by the European Center for Disease Control and Prevention (ECDC) revealed that *S. aureus* is the second most commonly isolated microorganism after *E. coli*, and it remains the leading cause of surgical site infections, while MRSA proportion varies greatly by country [4]. The World Health Organization’s global report on antibiotic resistance surveillance provides a global picture of MRSA expansion [5,6]. Despite the fact that systematic antibiotic resistance data were only available in developed countries in Europe and also in America, and Australia, etc., MRSA was reported on all continents. The prevalence of MRSA in most nations exceeded 20%, and in some cases reached 80%.

The MDR *Staphylococcal* infection is also becoming a growing concern in Bangladesh. This increased resistance is caused by inadequate hospital administration, a lack of understanding, and cross resistance, which leads to major health consequences. One study in Chittagong, Bangladesh found that 21 of 30 *S. aureus* bacteria isolates from pus, two out of four from blood, and three out of four from miscellaneous materials were MDR [7]. The study also revealed that the majority of them were medication resistant, with respect to ciprofloxacin (38.06%), cefradine (32.27%) and ampicillin (5.26%) sensitivity. Another study reported that 85.11% of MRSA samples collected in Dhaka, Rajshahi, Mymensingh, and Chittagong over a 12-month period were resistant to penicillin and ampicillin [8].

Several mechanisms, ranging from drug target alteration to active efflux pump systems, are involved in the development of this resistance. Numerous studies have been conducted in recent years to determine the underlying reasons behind antibiotic resistance so that appropriate interventions can be taken. The MDR efflux pumps are either chromosomal or plasmid-encoded and are responsible for developing resistance to various antimicrobial drugs, as well as the selection of drug resistant strains, have received special attention. These pumps, alone or in combination with other pumps, may act as a first line of defense against antimicrobials, allowing the cells to survive and thereby become antibiotic resistant [9]. Due to their promiscuous substrate specificity, the overexpression of MDR efflux pumps can enhance resistance to numerous classes of antibiotics while also decreasing susceptibility to biocides shown to lead to low-level antimicrobial resistance and the formation of MDR phenotypes in clinical isolates [10]. For example, the overexpression of *mepA* and *norB* genes showed a pattern of resistance towards tetracyclines, fluoroquinolines, dyes and disinfectants [11]. A previous study identified the combinations of these genes as being in charge of ciprofloxacin resistance [12]. Aside from the risk of therapeutic failure, the emergence of these multidrug resistant phenotypes could lead to other issues, such as possible co-selection and cross-resistance between efflux mediated antibiotic and biocide resistance, which is especially concerning for drug-resistant strains such as multidrug-resistant *S. aureus.*

Therefore, the present study was intended to find the association between the susceptibility profile of the clinical isolates and the prevalence of efflux pumps genes among them. The study will open up new avenues for future research on efflux pump encoding genes, efflux pumps, and their relationship with MDR. In addition, a new therapeutic concept based on the use of efflux pump inhibitors as adjuvants to conventional therapy could overcome the scarcity of therapeutic alternatives for multidrug resistance (MDR), as well as to help restore the activity of older and less expensive antibiotics.

## 2. Materials & Methods

### 2.1. Chemicals

Muller-Hinton agar and Muller-Hinton broth media were collected from TM media Ltd. of India. Amoxicillin (AMX 30 mg), ciprofloxacin (CIP 5 mg), cefixime (CFM 5 mg), tetracycline (TE 30 mg), chloramphenicol (C 30 mg), linezolid (LNZ 10 mg), azithromycin (AZM 15 mg), kanamycin (K 30 mg), vancomycin (VA 30 mg), and imipenem (IPM 10 mg) standards were generously donated by Advanced Chemical Industries Ltd., Dhaka, Bangladesh. The Promega DNA extraction kit was purchased from local suppliers. Specific primers and GoTaq^®^ G2 Green Master Mix were purchased from Biotech Concern Ltd., Bangladesh.

### 2.2. Bacterial Strain Collection

All of the experiments were performed according to the Clinical Laboratory Standards Institute (CLSI) guidelines (M100, 30th Ed. 2020) and the European Committee on Antibiotic Susceptibility Testing (EUCAST) for *Staphylococcus aureus* V. 9.0. A total of sixty clinical isolates of *Staphylococcus aureus* were collected from the Department of Microbiology and Immunology Laboratory; Bangabandhu Sheikh Mujib Medical University, Bangladesh from June 2021 to September 2021.

### 2.3. Bacterial Identification

Clinical isolates of *S. aureus* were identified and authenticated by the standard protocol developed by the collection center. Molecular investigation was carried out for further confirmation of the *S. aureus* bacteria by PCR using specific primers (Forward primer—5′-AGAGTTTGATCCTGGCTCAG-3′ and Reverse primer—5′-TACGGTTACCTTGTTACGACTT-3′ constructed from the 16S *r*RNA region of *S. aureus*. MRSA strains were identified by molecular identification of the *mecA* gene by PCR.

### 2.4. Bacterial Cell Culture

Sterile tubes containing sterilized MHA were inoculated with a pure culture of an identified clinical isolate of *S. aureus* under a laminar airflow (LAF) bench and were incubated at 37 ± 2 °C for 2–3 h.

### 2.5. Antibacterial Susceptibility Test

An antibacterial susceptibility test was performed by using the Kirby-Bauer disc diffusion technique for ten antibiotics mentioned earlier.

MHA and MHB were prepared and sterilized using the appropriate guidelines. Clinical isolates cultured in the MHA media were again inoculated into the test tubes containing MHB and were placed into a shaker incubator for 2 h at a temperature of 37 °C. The bacterial growth was adjusted to 10^5^ CFU/mL for 0.5 McFarland standard by adding phosphate buffer saline. Sterile MHA was poured into petri dishes and after the sterility of the plates was confirmed, bacteria were inoculated into the petri dishes with a cotton swab. The antibiotic discs were placed in the plates with sterilized forceps and then the plates were incubated upside down at 37 °C for 16–18 h. Post incubation, clear zones of inhibition (ZOI) were formed around the discs. The diameter of the zones was measured and interpreted in accordance with the Clinical and Laboratory Standards Institute (CLSI) antimicrobial susceptibility testing standards.

### 2.6. Amplification of Target Genes by Polymerase Chain Reaction

The extraction of total genomic DNA was done using a Wizard Genomic DNA Purification Kit. Specific primers for *norA, norB, norC, mepA, sepA, mdeA, qacA/B, smr* were used for the amplification (Table 1). An amount of 10 µL of PCR mixture was prepared by using 5 µL of GoTaq^®^ G2 Green Master Mix, 0.5 µL of forward primer, 0.5 µL of reverse primer, and 3 µL of nuclease-free water with 1.0 µL of template DNA. A negative control without template DNA was also used.

The presence of chromosomally-encoded and plasmid-encoded efflux pump genes was examined in all of the isolated strains. For chromosomally-encoded efflux genes, the initial denaturation was set at 94 °C for 4 min, followed by 35 cycles of denaturation at 94 °C for 30 s. The annealing temperature was 57–60 °C (varied with genes) for 55 s, extension at 72 °C for 1 min, and a final extension of 72 °C for 5 min. For plasmid-encoded efflux genes, the initial denaturation was set at 95 °C for 1 min followed by 30 cycles of denaturation at 95 °C for 30 s, an annealing temperature of 56–58 °C for 45 s, extension at 72 °C for 1 min, and a final extension of 72 °C for 5 min. For the *mecA* gene, initial denaturation was set at 94 °C for 4 min followed by 35 cycles of final denaturation at 95 °C for 30 s. The annealing temperature was 53 °C for 30 s. An extension was conducted at 72 °C for 1 min with a final extension of 72 °C for 5 min.

The amplification products of the desired genes were visualized by resolving the PCR products in 0.6% agarose gel at 80 volts and 100 mA for 40 min. The gel was viewed using the Alpha Imager HP Gel documentation system (Cell Bioscience, Santa Clara, CA, 95051, USA), photographs were taken using a computer attached to the machine, and the bands were analyzed.

## 3. Result

### 3.1. Antibacterial Susceptibility Pattern in Clinical Isolates of S. aureus

**Figure 1:**
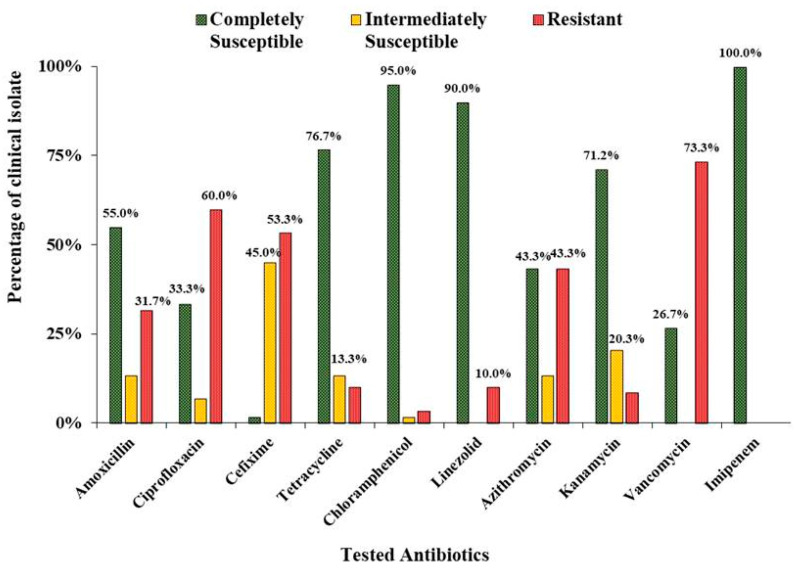
Antibacterial susceptibility profile of the clinical isolates of *S. aureus*.

In the present study, antibacterial sensitivity was tested using the Kirby-Bauer disc diffusion method to detect multidrug-resistant *S. aureus* among the collected clinical isolates. The clinical insolates of *Staphylococcus aureus* were evaluated for their susceptibility towards ten different antibiotics of different groups (Figure 1). A higher number of *S. aureus* showed resistance to vancomycin (73.33%), followed by ciprofloxacin (60%), cefixime (53.33%), azithromycin (43.33%), and amoxicillin (31.67%). However, the lower number of isolates showed resistance against chloramphenicol (3.33%), kanamycin (8.47%), linezolid (10%), and tetracycline (10%). Imipenem, on the other hand, turned out to be fully effective against the tested antibiotics. Among the isolates, 55% showed resistance to three or more classes of drugs, thereby meeting the classical definition of multidrug resistance (SM’s are enclosed herewith).

### 3.2. Characterizations of Different Types of S. aureus in Clinical Isolates

Different kinds of *S. aureus* have been found among the clinical isolates worldwide. Molecular characterization with PCR targeting the presence of the *mecA* gene, which is the hallmark of methicillin resistant *S. aureus*, was used to identify the presence of this bacteria among the samples. Of 60 isolates, 60% turned out to be MRSA, as they contained the *mecA* gene, which is responsible for methicillin resistance in *S. aureus*. If a clinical isolate showed resistance to at least three categories of different antibiotics, it is speculated to be multi-drug resistant. According to the susceptibility profile carried out with ten different antibiotics, a total of 33 clinical isolates of *S. aureus* showed resistance against at least three different antibiotics (Table 2). Among the MSSA (*n* = 24) clinical isolates, 15 samples were shown to have MDR characteristics, whereas 18 MRSA samples were found to be multi-drug resistant.

### 3.3. Prevalence of Different Efflux Pump Genes in the Clinical Isolates of S. aureus

**Figure 2:**
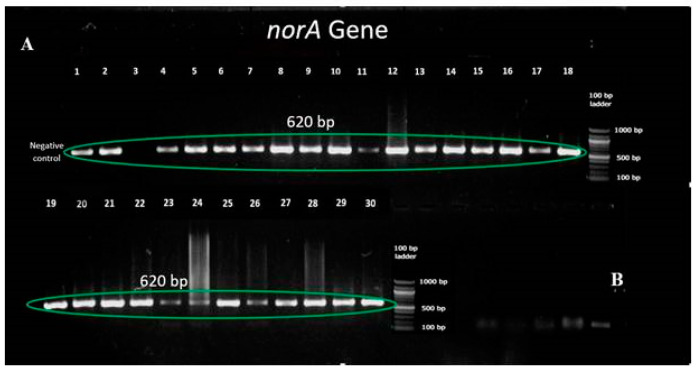
(**A**,**B**) Detection of *norA* gene in the clinical isolates using PCR and gel electrophoresis.

Efflux pump genes were identified using polymerase chain reaction and were visualized by agarose gel electrophoresis. A sample representing the presence of *norA* gene in different clinical isolates has been shown below (Figure 2). 

**Figure 3:**
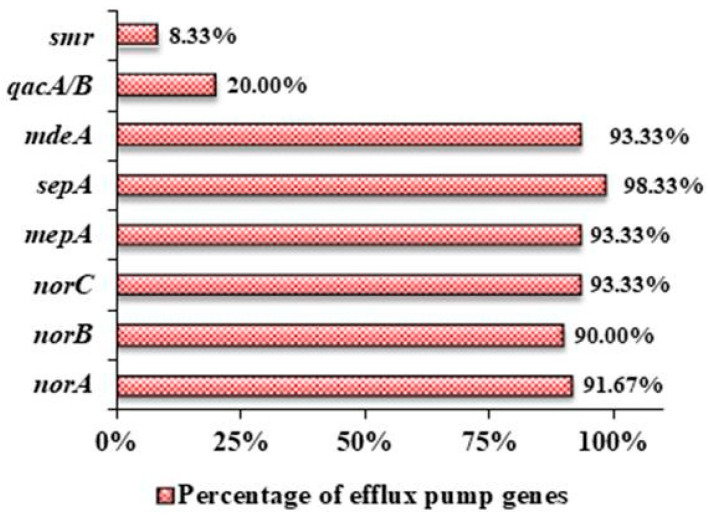
Prevalence of different efflux pump genes in the clinical isolates of *S. aureus*.

An extremely high prevalence of chromosomally encoded genes including *norA* (91.67%), *norB* (90%), *norC* (93.33%), m*epA* (93.33%), s*epA* (98.33%), and *mdeA* (93.33%) was observed among the clinical isolates (*n* = 60), while very few of them contained plasmid-encoded genes *qacA/B* (20%) and *smr* (8.33%) as well (Figure 3).

**Figure 4:**
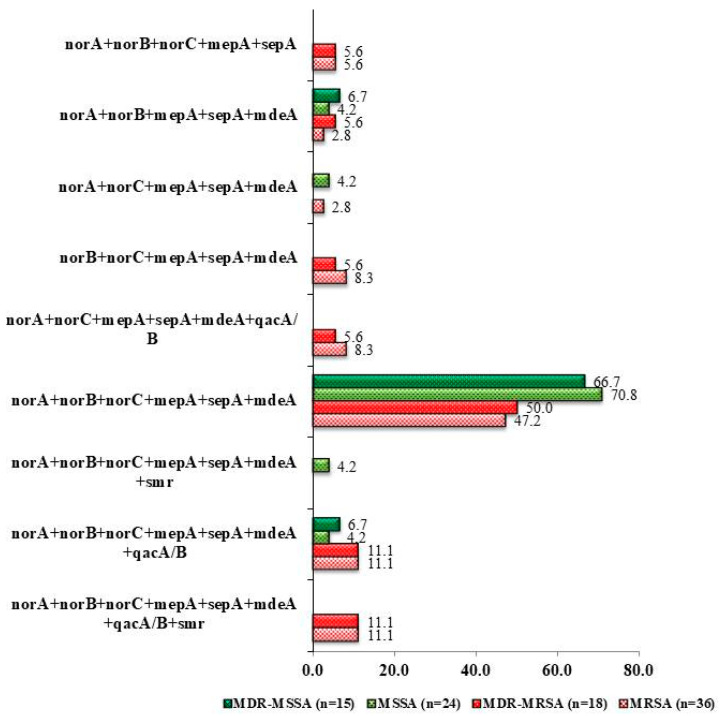
Prevalence of different combinations of genes present in the collected clinical isolates of *S. aureus.*

Irrespective of their resistance status, the most effective combination of efflux pump genes was chromosomal encoded *norA + norB + norC + mepA + sepA + mdeA* among the clinical isolates including MRSA and MSSA, which provided a higher prevalence of resistance against the tested antibiotics (Figure 4). Six chromosomal genes were found to be 47.2% and 70.8% in methicillin-resistant *S. aureus* (MRSA) and methicillin-sensitive *S. aureus* (MSSA) clinical isolates, respectively. Two plasmid encoded genes such as *qac*A/B and *smr* along with chromosomal encoded genes were present in only MDR MRSA (11.1%). However, *qacA/B* along with six chromosomal encoded genes was found in both MDR MRSA (11.1%) and MDR MSSA (4.2%) isolates (Figure 4).

### 3.4. Antibiotic Resistant Profile Associated with Efflux Pump Genes Combination

In the present study, the profiling of antibiotic resistant genes in the clinical isolates of *S. aureus* was investigated. The presence of at least three genes encoding efflux pumps in those clinical isolates of *S. aureus* that have largely showed resistance to several antibiotics was observed. More than six combinations of genes were found in the clinical isolates that showed resistance to five different antibiotics, namely vancomycin, amoxicillin, ciprofloxacin, cefixime, and azithromycin. The details of the different combinations of genes with respect to various antibiotic resistant isolates are shown in Table 3 and Table 4.

## 4. Discussion

This study was conducted on both methicillin resistant *S. aureus* (MRSA) and methicillin sensitive *S. aureus* (MSSA) collected from a tertiary hospital in Bangladesh. Based on our result, this is the first report in Bangladesh on the detection of several kinds of efflux pump genes in the clinical isolates of *S. aureus*. It enabled us to get a clear perception of the effectivity of different classes of antibiotics against *S. aureus* and to detect the prevalence of specific efflux protein encoding genes with a view to establishing a possible association between efflux pumps and multidrug resistance in *S. aureus.*

The clinical isolates were classified as methicillin-sensitive *S. aureus*, methicillin-resistant *S. aureus*, MDR methicillin-sensitive *S. aureus*, and MDR methicillin-resistant *S. aureus,* where 60% of isolates turned out to be MRSA, which became a potential threat to the patients compared to MSSA [13,14]. Parvez et al. 2018 [15] reported a 72% prevalence of MRSA in the clinical isolates depending upon the presence of the *mecA* gene, which had a higher incidence rate than that indicated in another report from Bangladesh published in 2005 [8]. On the other hand, our findings are similar with a published report on the clinical samples of *S. aureus* collected from different hospitals of Chittagong, where they noted that 65.15% were of the MRSA strain [16]. Compared with a previous report from Western Europe where MRSA from the clinical isolates of *S. aureus* was found in a range between 5 to 54% [17], Bangladeshi isolates are more prone to be identified as MRSA.

In the present study, 98.33% of bacterial samples showed resistance against at least one class of drug. However, all of the strains showed complete susceptibility towards imipenem, a carbapenem derivative, which matched with the previous report from another hospital in Bangladesh [18]. Although the same report showed that all isolates were sensitive to vancomycin, we observed a significant difference here because 73.3% of isolates were resistant to it. It is evident that all of them can be designated as vancomycin resistant *S. aureus* strains (VRSA), which was first reported in the U.S. in 2002 [19]. Vancomycin resistance in the bacteria is generally conferred by the *vanA* operon encoded on Transposon Tn*1546* in the Vancomycin resistant Enterococci (VRE), which may horizontally transfer to *S. aureus* isolates in our study isolates [20]. However, it is the first report of observing VRSA strains in Bangladesh which makes it a serious concern for clinicians. Surprisingly, a significant portion of the clinical isolates showed sensitivity towards amoxicillin compared to cefixime in this study (Figure 1), despite the latter antibiotic belonging to a more advanced class of beta-lactam antibiotics.

The extrusion of multiple substrates through efflux pump proteins being a predominant mechanism of antibacterial resistance, eight of the well-studied genes responsible for expressing different efflux proteins in *S. aureus* were taken into consideration and were used to find out whether there is any possible common genetic combination among the collected clinical isolates that may confer higher antibiotic resistance. It was found that irrespective of their resistance pattern, each bacterial sample contained at least four efflux protein-encoding genes in their DNA (Table 3). While the individual percentage of the prevalence of chromosomally-encoded genes exceeded an average of 90%, very few possessed plasmid-encoded efflux protein genes, with the *smr* gene being the least prevalent (8.33%). While several studies have been conducted individually on MFS transporter *norA* [21], *norB* [22], *norC* [23], *mepA* [24], *sepA* [25], and *mdeA* [26], as well as on their role in ciprofloxacin resistance, our findings are in accordance with these aforementioned works. A systematic review on the epidemiology of efflux pump genes on *S. aureus* also revealed similar findings, where *norA* (75%) was highly reported in Asia, and the least reported was *qacA/B* (20.8%) [27]. Table 5 shows a comparison of the prevalence of efflux pump genes in the clinical isolates of different countries.

All 21 ciprofloxacin-resistant MRSA isolates had at least five chromosomal encoded genes. However, a combination of six genes such as *norA* + *norB* + *norC* + *mepA* + *sepA* + *mdeA* was found to be widely distributed among the clinical samples. All of these, except *sepA,* were found predominantly in the Iraqi patients as well [29]. Seven of the 21 ciprofloxacin-resistant MRSA contained at least one plasmid-encoded gene *qacA/B* and only two of them had *smr* gene along with six chromosomal encoded genes. A similar result was reported in African countries, where plasmid encoded *qacA/B* (40.5%) and *smr* (3.7%) genes were reported from the *S. aureus* isolates [34]. The presence of plasmid encoded *qacA/B* genes in the MRSA showed complete resistance towards ciprofloxacin and cefixime. This observation hinted toward a potentiated action of efflux pumps due to the presence of multiple efflux proteins in the same clinical isolate.

A similar distribution of genes was observed in the amoxicillin, cefixime, azithromycin, and vancomycin-resistant bacterial samples as well, since they possessed various genetic combinations, with the major portion containing at least six efflux genes in their DNA. However, whether these efflux pumps are the primary cause of developing resistance against other classes of drugs or not remains to be investigated. It is to be noted that efflux pumps are generally responsible for developing resistance against ciprofloxacin, tetracycline and azithromycin.

It has been documented that *mecA* gene is responsible for encoding the low-affinity penicillin-binding protein PBP 2A that confers methicillin resistance in *S. aureus* [35]. Among the amoxicillin-resistant strains, 57.89% of samples had the *mecA* gene, whereas the percentage was higher in cefixime-resistant strains (68.75%). The greater distribution of the *mecA* gene in cefixime compared to amoxicillin made us wonder about a shift in their evolutionary process, since bacterial cells stop undergoing mutational changes in the absence of antibiotic molecules, as doing otherwise adversely affects cellular homeostasis. Hence, the lower distribution of *mecA* in amoxicillin-resistant clinical strains and their improved susceptibility towards penicillin could be attributed to its lesser use in clinical practice compared to the other classes of antibiotics. The same inference can be made in the case of tetracycline, chloramphenicol and linezolid, because most of the isolates showed susceptibility towards these antibiotics in this study. If this is indeed the case, then it could make a significant contribution to the field of medicine in the field of antibiotic resistance. Nevertheless, the coexistence of the *mecA* gene with multiple efflux proteins in resistant bacterial strains made us ponder their synergistic effect. However, MSSA isolates possessing similar combinations of chromosomal genes exhibited a similar antibiotic resistance pattern when compared with the MRSA isolates. Notably, the combination of *norA* + *norB* + *norC* + *mepA* + *sepA* genes presenting in MSSA isolates were shown to be completely resistant to ciprofloxacin, azithromycin, and vancomycin, which shifted our focus to the expression of these genes and the specific conditions favoring it.

## 5. Conclusions

Antibiotic resistance is a worldwide phenomenon. With its constantly evolving molecular mechanism, *Staphylococcus aureus* has become a major catalyst behind this global health hazard. To combat it, we first need to identify the resistance pattern of this pathogen and the mechanisms responsible for its resistance. In this study, we found some common patterns of resistance and identified a number of clinically significant genes. However, our study had some limitations as well, since we have only observed the prevalence of specific genes but have not examined their expression in the clinical isolates. Hence, further study is needed to determine the association between efflux proteins and the waning sensitivity of *S. aureus* to different classes of drugs. However, based on the findings of our study, we could conclude that the resistance pattern of *S. aureus* in the Bangladeshi population is largely attributable to the prevalence of different efflux proteins.

## Figures and Tables

**Table 1:** List of primers with detailed information for the identification of different efflux pump genes.

Primers		Oligonucleotide Sequence	%GC Content	Tm (°C)	Amplicon Size
*norA*	Forward	5′-TTCACCAAGCCATCAAAAAG-3′	40	54.3	620 bp
	Reverse	5′-CTTGCCTTTCTCCAGCAATA-3′	45	56.4	
*norB*	Forward	5′-AGCGCGTTGTCTATCTTTCC-3′	50	58.4	213 bp
	Reverse	5′-GCAGGTGGTCTTGCTGATAA-3′	50	58.4	
*norC*	Forward	5′-AATGGGTTCTAAGCGACCAA-3′	45	56.4	216 bp
	Reverse	5′-ATACCTGAAGCAACGCCAAC-3′	50	58.4	
*mepA*	Forward	5′-ATGTTGCTGCTGCTCTGTTC-3′	50	58.4	718 bp
	Reverse	5′-TCAACTGTCAAACGATCACG-3′	45	56.4	
*sepA*	Forward	5′-GCAGTCGAGCATTTAATGGA-3′	45	56.4	103 bp
	Reverse	5′-ACGTTGTTGCAACTGTGTAAGA-3′	41	58.4	
*mdeA*	Forward	5′-AACGCGATACCAACCATTC-3′	47	55.2	677 bp
	Reverse	5′-TTAGCACCAGCTATTGGACCT-3′	48	59.4	
*qacA/B*	Forward	5′-GCTGCATTTATGACAATGTTTG-3′	36	56.6	628 bp
	Reverse	5′-AATCCCACCTACTAAAGCAG-3′	45	56.4	
*smr*	Forward	5′-ATAAGTACTGAAGTTATTGGAAGT-3′	29	56.7	285 bp
	Reverse	5′-TTCCGAAAATGTTTAACGAAACTA-3′	29	56.7	
*mecA*	Forward	5′-GTTGTAGTTGTCGGGTTTGG-3′	50	58.4	331 bp
	Reverse	5′-CTTCCACATACCATCTTCTTTAAC-3′	38	60.1	

**Table 2:** Characterization of different types of *S. aureus* in the clinical isolates (*n* = 60).

Characterizations of Isolates	No. of Isolates	Percentage (%)	Characterizations of Isolates	No. of Isolates	Percentage (%)
Methicillin resistant *S. aureus* (MRSA)	36	60%	MDR-MRSA	18	30%
			Regular-MRSA	18	30%
Methicillin sensitive *S. aureus*(MSSA)	24	40%	MDR-MSSA	15	25%
			Regular-MSSA	9	15%
Multi-drug resistant *S. aureus*	33	55%	Regular *S. aureus*	27	45%

**Table 3:** Cumulative frequency of different combinations of efflux pump genes in different clinical isolates.

Status of Isolates	No. of Isolates	4 Genes	5 Genes	6 Genes	7 Genes	8 Genes
MRSA	36	36	35	27	7	3
MDR MRSA	18	-	18	14	4	2
MSSA	24	24	22	19	2	-
MDR MSSA	15	15	13	11	1	-

**Table 4:** Frequency of different combinations of efflux pump genes against different antibiotic resistant clinical isolates.

Gene Combinations	AMX	CIP	CFM	AZM	VAN
**MRSA (*n* = 36)**					
Resistant isolates (*n*)	11	21	22	13	25
*norA* + *norB* + *norC* + *mepA* + *sepA* + *mdeA*	3	9	8	6	13
*norA* + *norB* + *norC* + *mepA* + *sepA* + *mdeA* + *qacA/B*	2	2	4 *	1	2
*norA* + *norB* + *norC* + *mepA* + *sepA* + *mdeA* + *qacA/B* + *smr*	-	2	3	2	2
*norA + norC + mepA + sepA + mdeA + qacA/B*	2	3 *	2	2	2
*norB + norC + mepA + sepA + mdeA*	2	3	2	1	2
*norA + norB + norC + mepA + sepA*	-	2 *	1	-	3 *
**MDR-MRSA (*n* = 18)**					
Resistant isolates (*n*)	10	14	15	9	17
*norA* + *norB* + *norC* + *mepA* + *sepA* + *mdeA*	5 *	9 *	7	4	9 *
*norA* + *norB* + *norC* + *mepA* + *sepA* + *mdeA* + *qacA/B*	1	2 *	2 *	2 *	2 *
*norA* + *norB* + *norC* + *mepA* + *sepA* + *mdeA* + *qacA/B* + *smr*	-	2 *	2 *	1	2 *
**MSSA (*n* = 24)**					
Resistant isolates (*n*)	8	15	13	10	19
*norA* + *norB* + *norC* + *mepA* + *sepA* + *mdeA*	5	11	6	7	13
*norA* + *norB* + *norC* + *mepA* + *sepA* + *mdeA* + *qacA/B*	1	-	1 *	1 *	1 *
*norB + norC + mepA + sepA + mdeA*	-	1 *	-	1 *	-
*norA + norB + norC + mepA + sepA*	-	2 *	1	2 *	2 *
**MDR-MSSA (*n* = 15)**					
Resistant isolates (*n*)	8	11	9	11	13
*norA* + *norB* + *norC* + *mepA* + *sepA* + *mdeA*	5	8	5	7	8
*norA* + *norB* + *norC* + *mepA* + *sepA* + *mdeA* + *qacA/B*	1	-	1 *	1 *	1 *
*norA + norB + norC + mepA + sepA*	-	2 *	1	2 *	2 *

* The combination of genes present in those isolates showed complete resistance to the respective antibiotic. AMX- amoxicillin, CIP-Ciprofloxacin, CFM-Cefixime, AZM-Azithromycin VAN-Vancomycin.

**Table 5:** Frequency of different efflux pump genes in *S. aureus* reported among different countries.

Countries	Efflux Pump Genes (%)								References
	norA	norB	norC	mepA	sepA	mdeA	qacA/B	smr	
China	96.2	98.2	92.5	90.6	96.2	94.3	83.0	77.4	[28]
Iraq	80.2	56.2	17.8	92.7	-	100	-	-	[29]
South Africa	98.9	98.9	79.4	97.9	96.9	95.9	-	-	[30]
Iran	41.7	41.7	41.7	60.0	35.0	61.7	3.3	30.0	[12]
Portugal	-	-	-	100	100	-	22.4	1.0	[31]
Malaysia	-	-	-	-	-	-	83.3	1.6	[32]
Canada	-	-	-	-	-	-	2.0	7.0	[33]

## Data Availability

The datasets originated in this study will be available upon request or in the Supplementary Files attached with this manuscript.

## Supplementary Materials

The following supporting information can be downloaded at: https://www.mdpi.com/article/10.3390/antibiotics12020305/s1.
