# Supplementary material for: Association of Antibacterial Susceptibility Profile with the Prevalence of Genes Encoding Efflux Proteins in the Bangladeshi Clinical Isolates of Staphylococcus aureus
Source: Antibiotics (Basel). 2023 Feb 2;12(2):305. doi: 10.3390/antibiotics12020305 (PMC9952083; doi:10.3390/antibiotics12020305)
Supplement: Supplementary file 1 [file antibiotics-12-00305-s001.zip › antibiotics-2180684-supplementary.pdf]

Association of antibacterial susceptibility profile with the prevalence of genes encoding efflux proteins in the Bangladeshi clinical isolates of *Staphylococcus aureus*

Tanjina Akter Suma,[1, †] Nushrat Alam,[1, †] Sheikh Zahir Raihan, [1]* Md. Al Zahid,[2] Shankar C. Mandal [2], Fahrin Jahan Suchana,[1] Ripa Kundu,[1] Anwar Hossain,[2] Md. Abdul Muhit [1]*

[1] *Department of Clinical Pharmacy and Pharmacology, Faculty of Pharmacy, University of Dhaka, Dhaka 1000, Bangladesh*

[2] *Department of Fisheries, University of Dhaka, Dhaka 1000, Bangladesh*

[†] *These authors contributed equally to this work*

* Author for Correspondence: **Dr. Md. Abdul Muhit**

Professor

Department of Clinical Pharmacy and Pharmacology

University of Dhaka, Bangladesh

E-mail: muhit@du.ac.bd

Phone: +88-01733-982854

Contents

1. Susceptibility Profiles of all Staphylococcus aureus bacteria: 3

2. Gel doc images of all genes.... 18

1. **Susceptibility Profiles of all Staphylococcus aureus bacteria:**

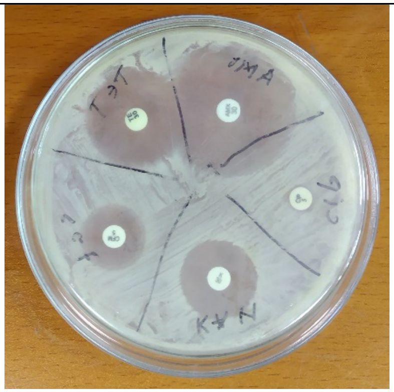	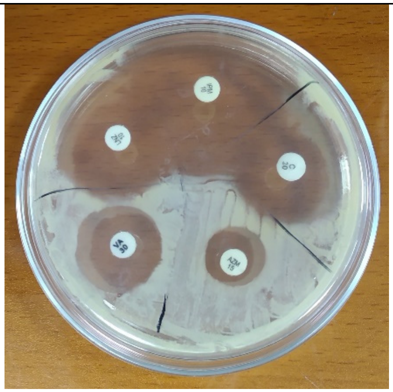	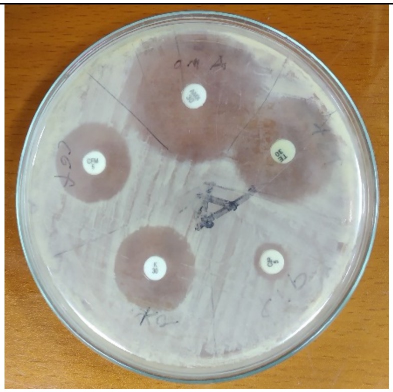	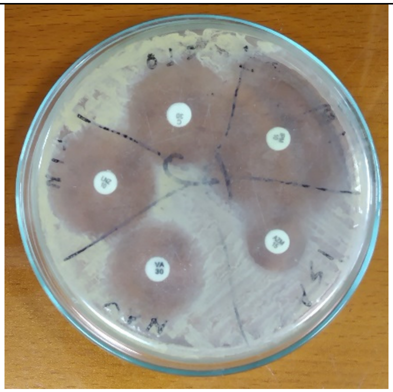
**Sample: 01**		**Sample: 02**	
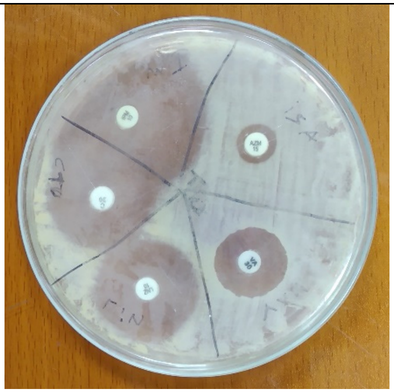	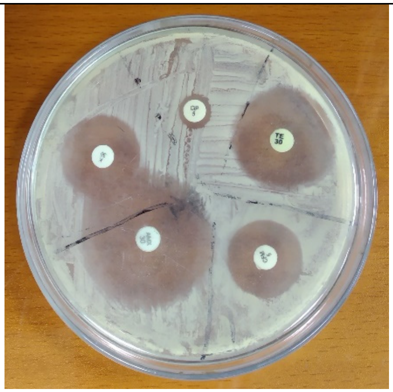	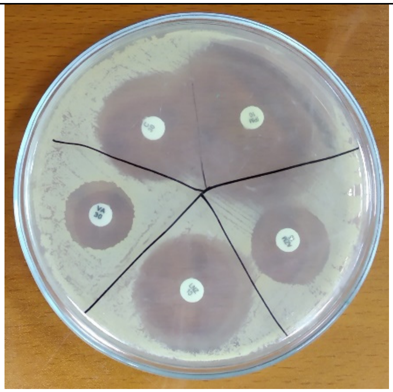	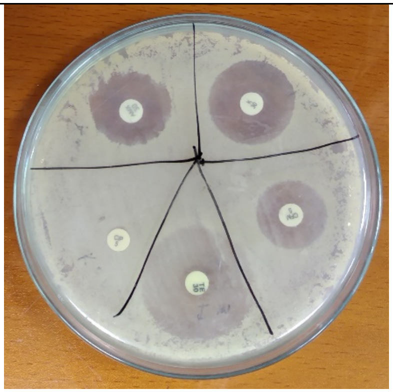
**Sample: 03**		**Sample: 04**	

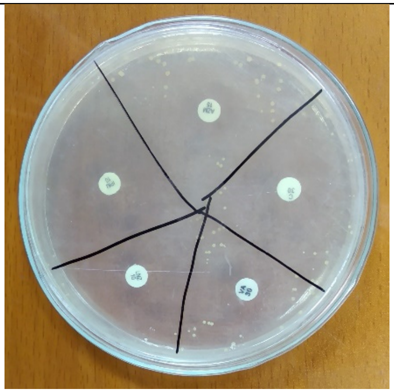	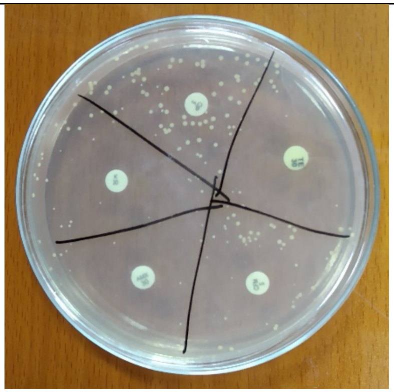	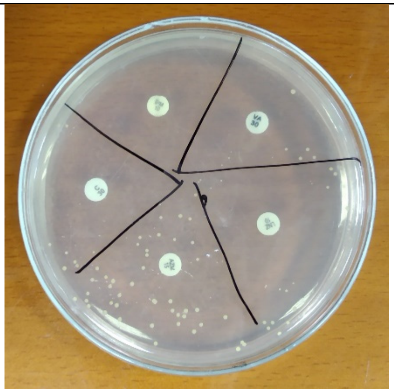	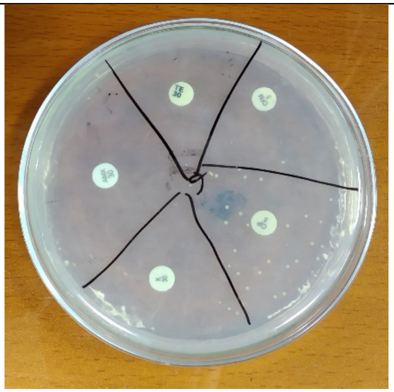
**Sample: 05**		**Sample: 06**	
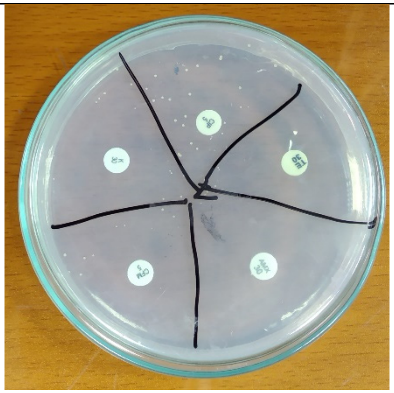	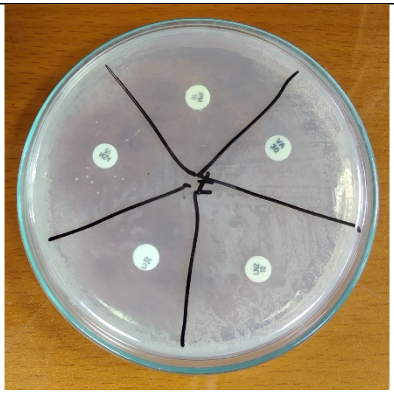	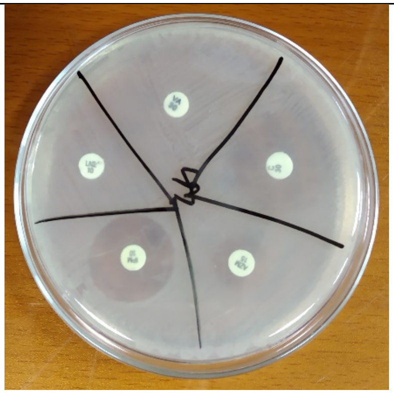	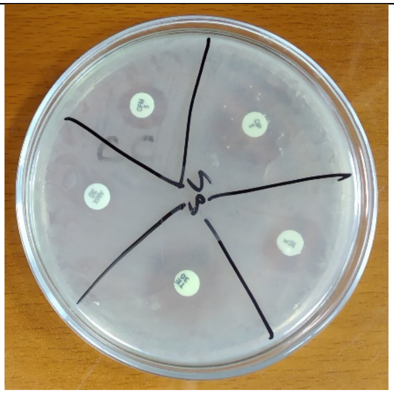
**Sample: 07**		**Sample: 08**	

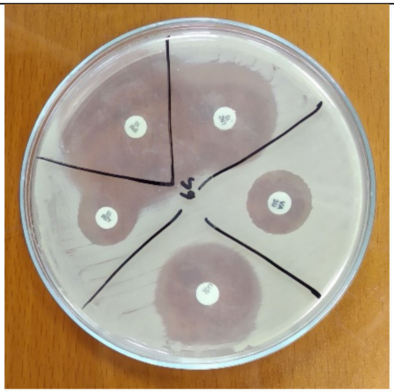	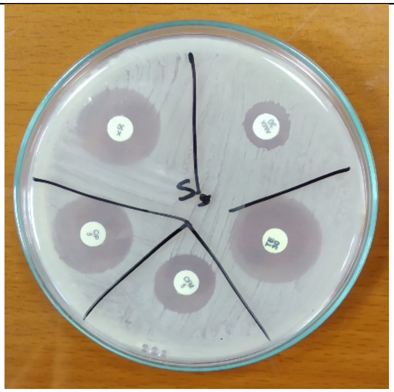	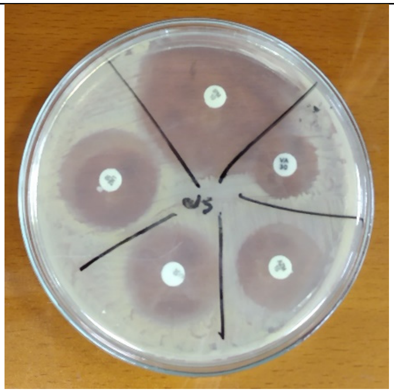	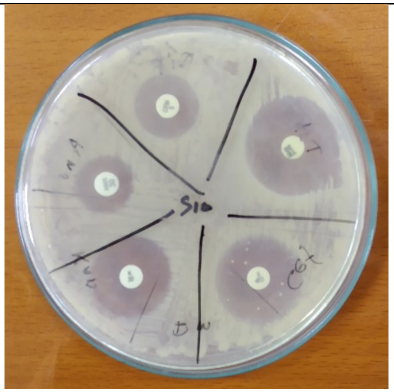
**Sample: 09**		**Sample: 10**	
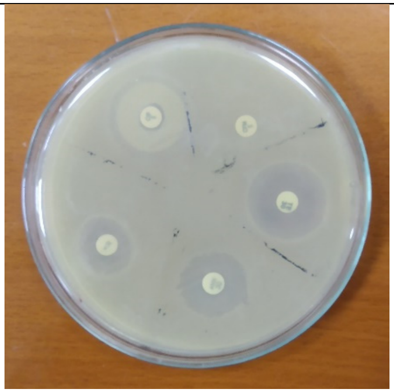	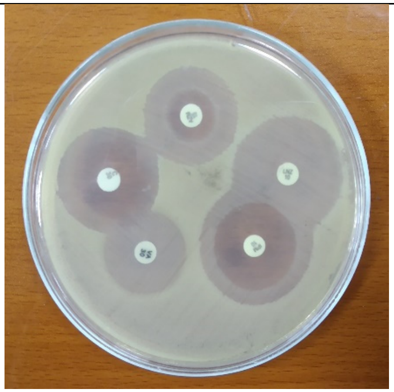	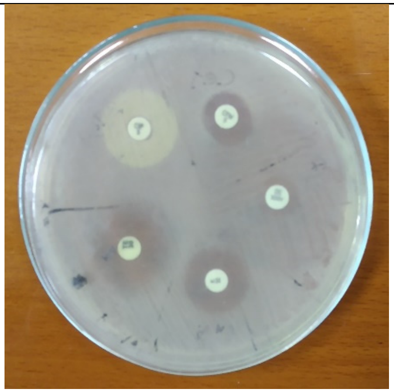	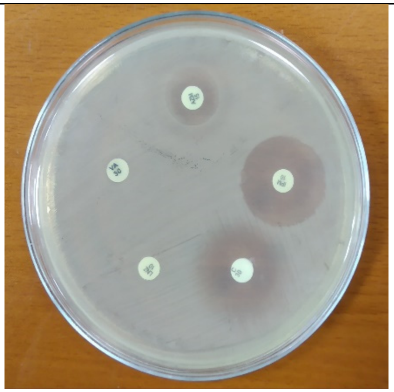
**Sample: 11**		**Sample: 12**	

<table>
<tr><td>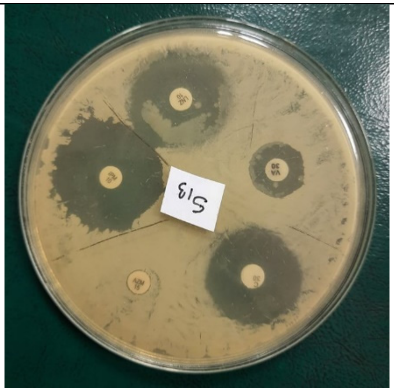</td><td>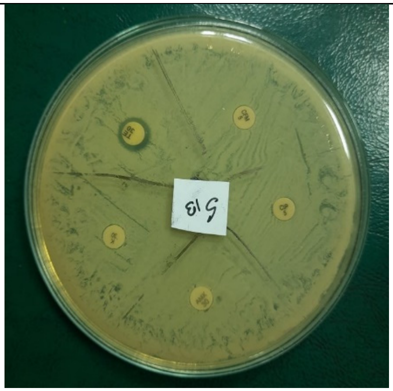</td><td>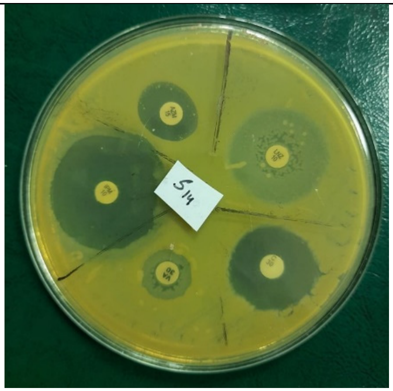</td><td>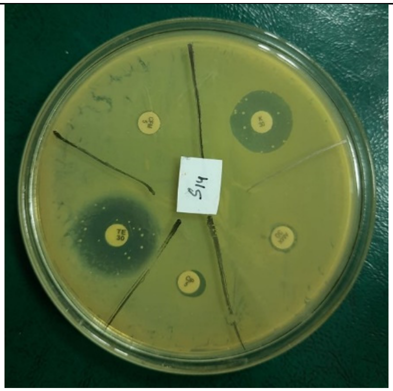</td></tr>
<tr><td colspan="2">Sample: 13</td><td colspan="2">Sample: 14</td></tr>
<tr><td>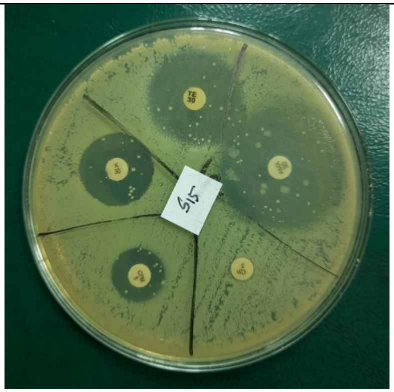</td><td>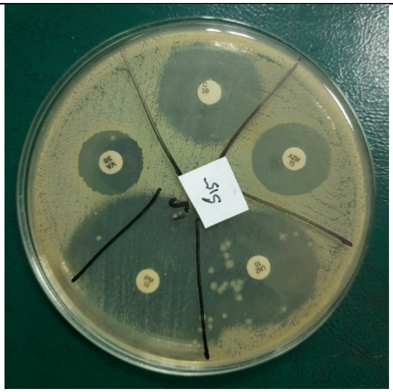</td><td>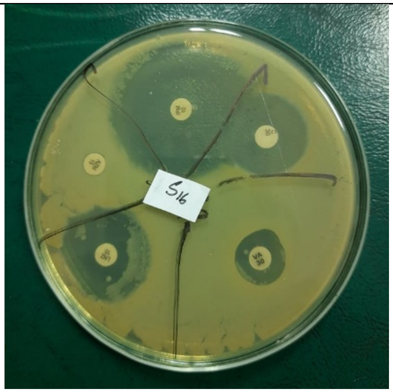</td><td>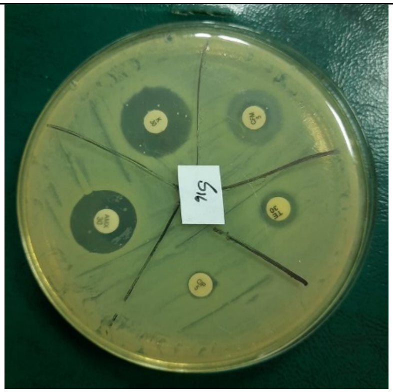</td></tr>
<tr><td colspan="2">Sample: 15</td><td colspan="2">Sample: 16</td></tr>
</table>

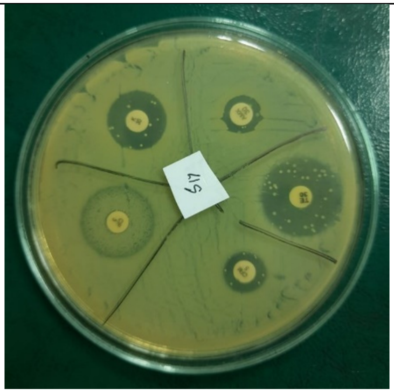	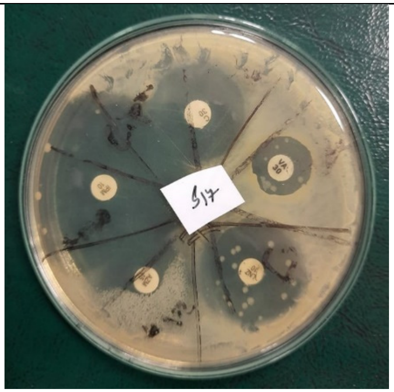	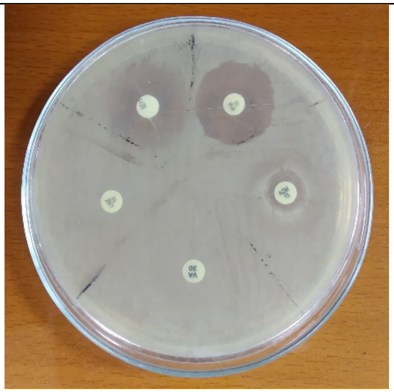	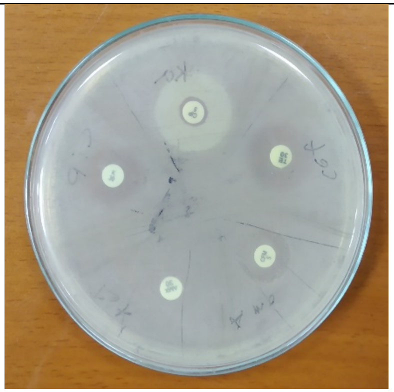
**Sample: 17**		**Sample: 18**	
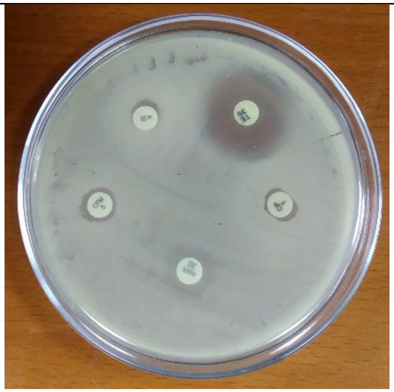	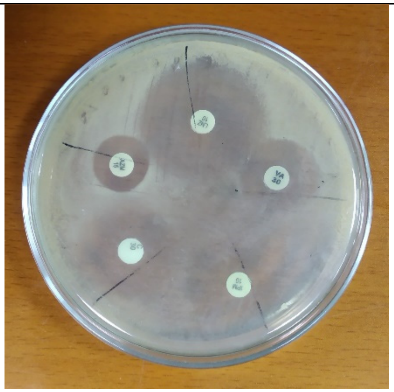	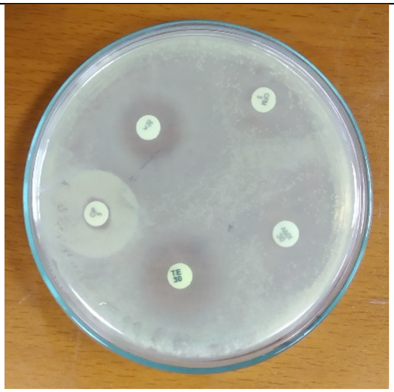	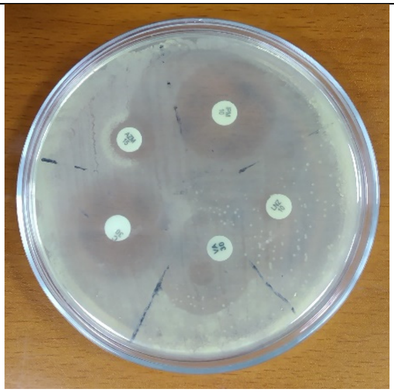
**Sample: 19**		**Sample: 20**	

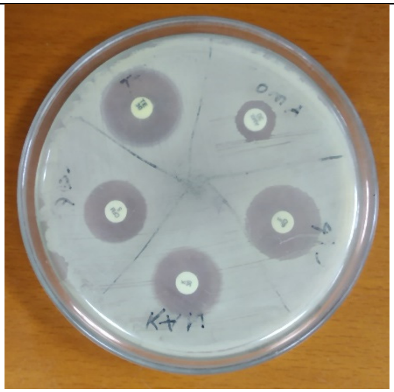
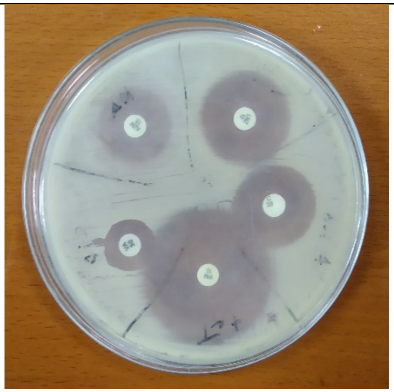

Sample: 21

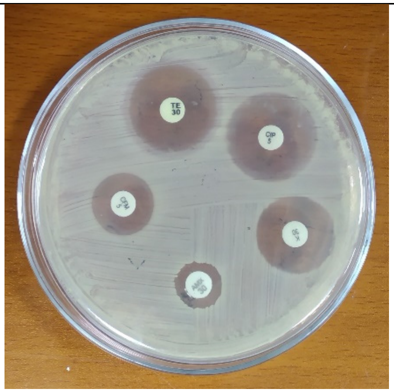
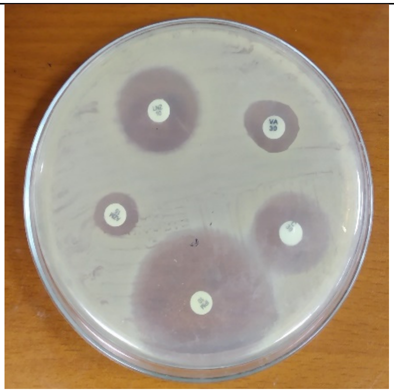

Sample: 22

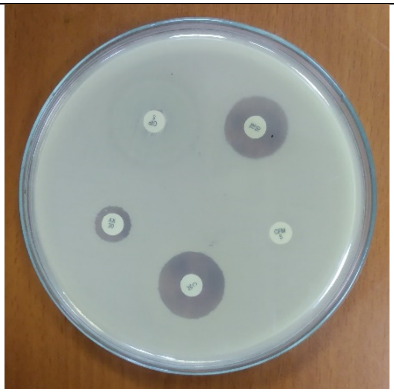
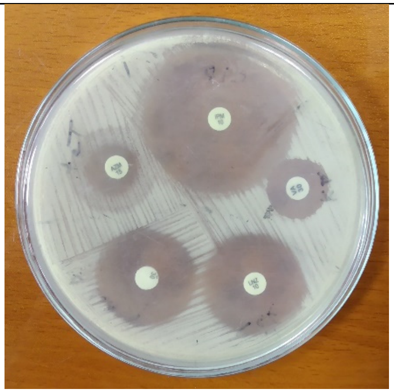

Sample: 23

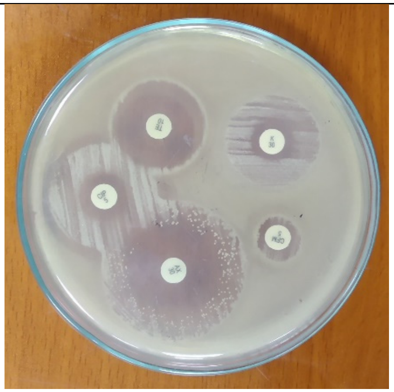
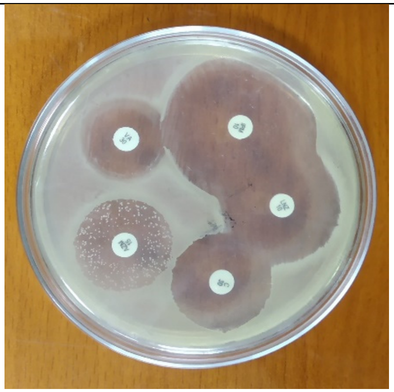

Sample: 24

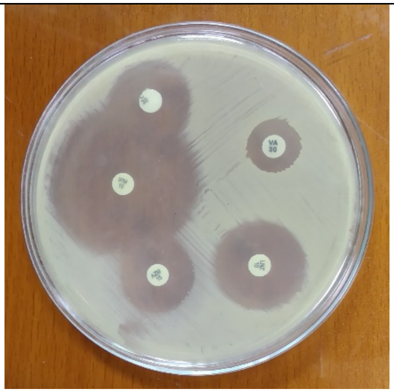 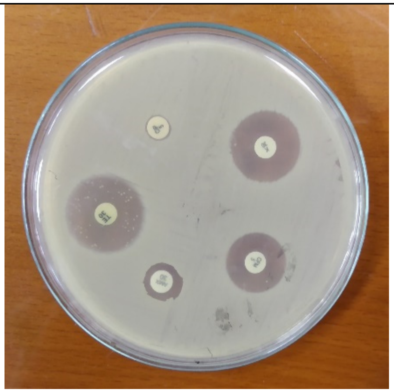

Sample: 25

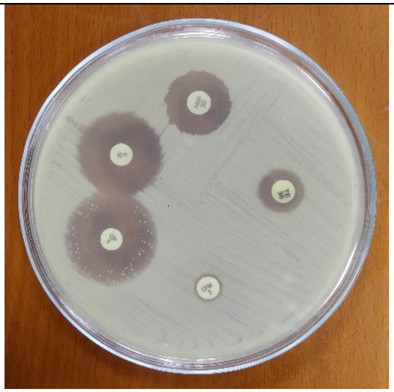 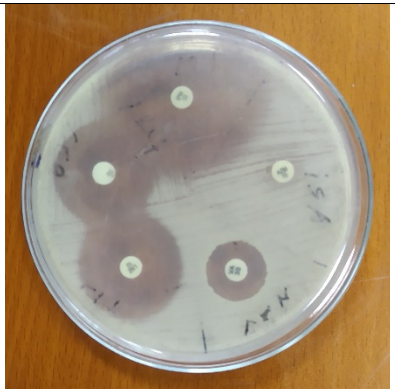

Sample: 26

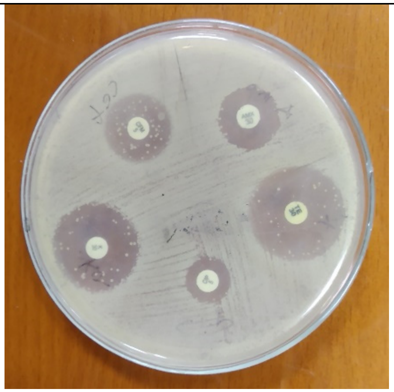 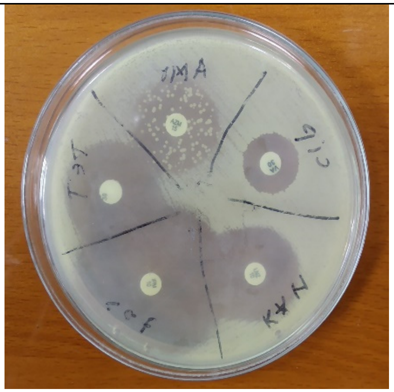

Sample: 27

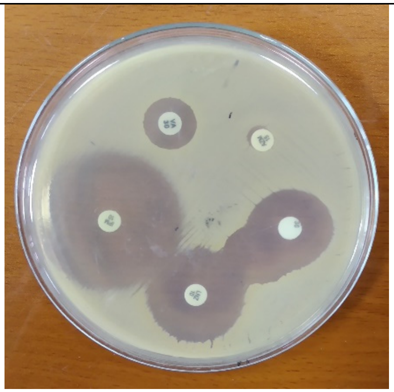 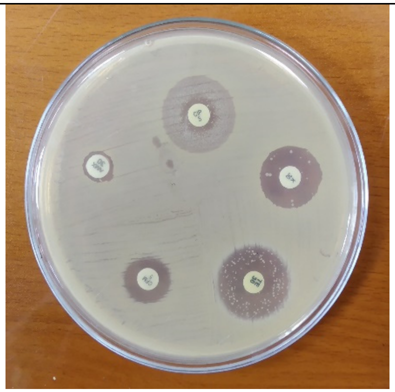

Sample: 28

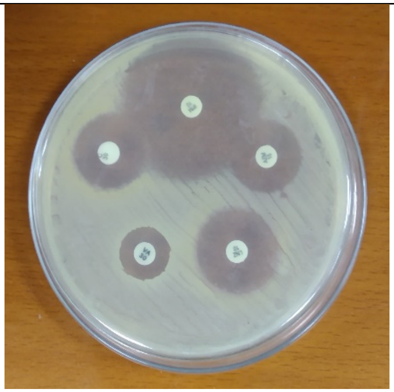	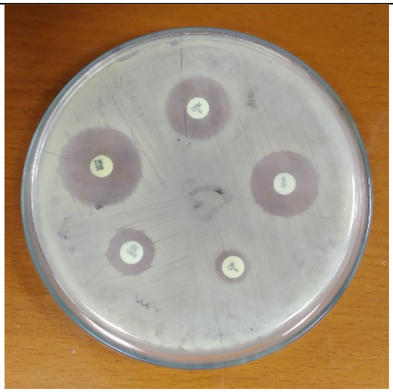	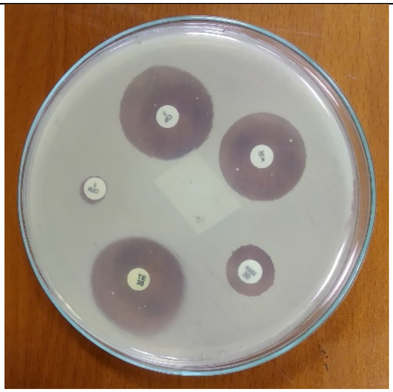	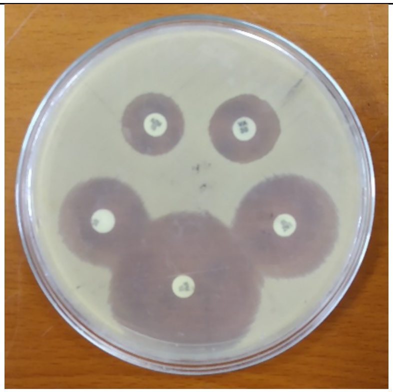
**Sample: 29**		**Sample: 30**	
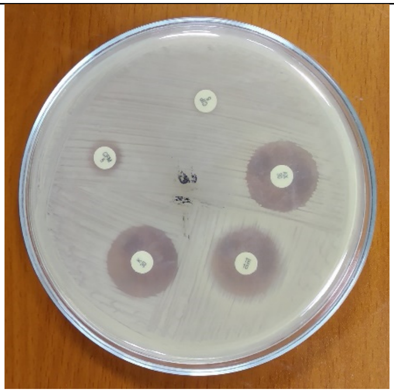	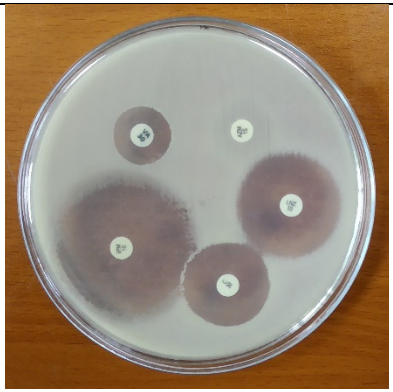	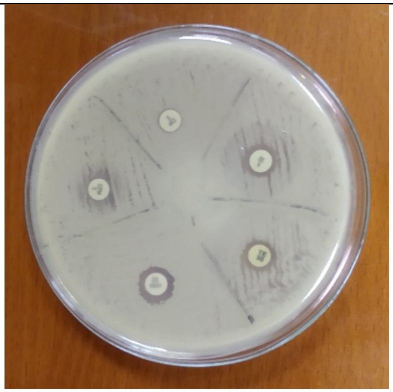	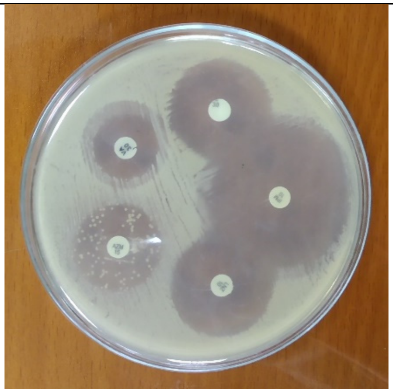
**Sample: 31**		**Sample: 32**	

<table>
<tr><td>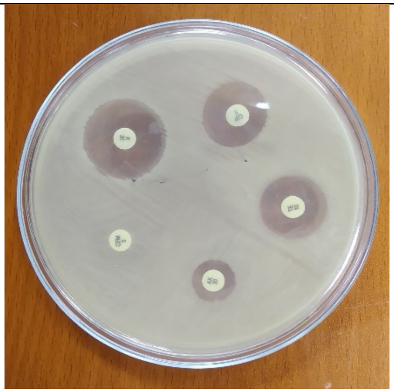</td><td>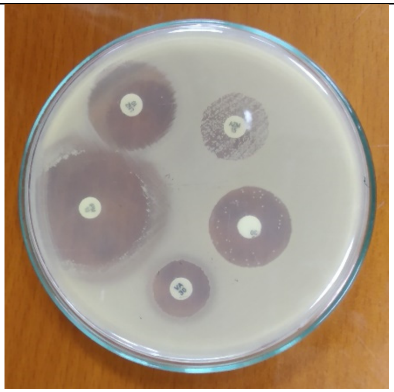</td><td>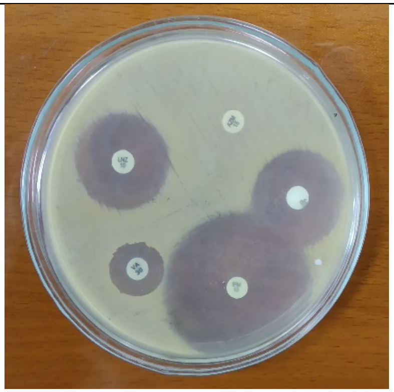</td><td>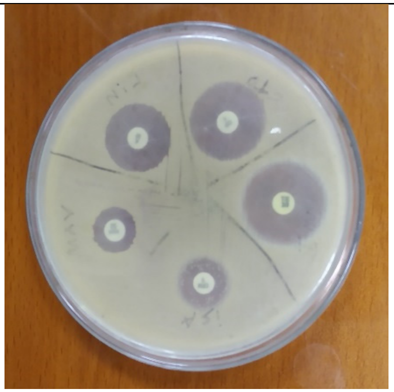</td></tr>
<tr><td colspan="2">Sample: 33</td><td colspan="2">Sample: 34</td></tr>
<tr><td>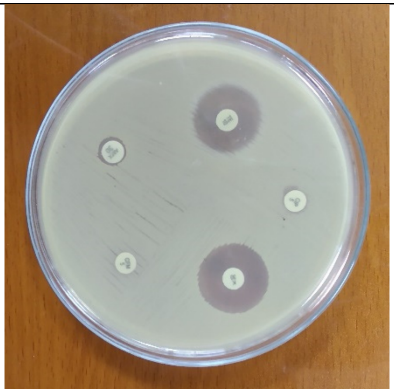</td><td>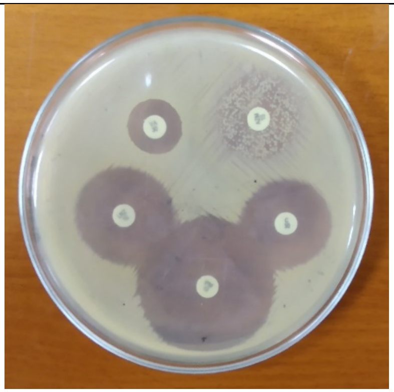</td><td>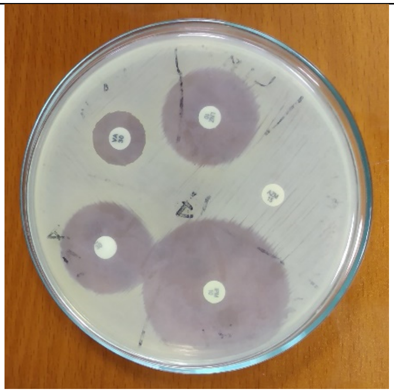</td><td>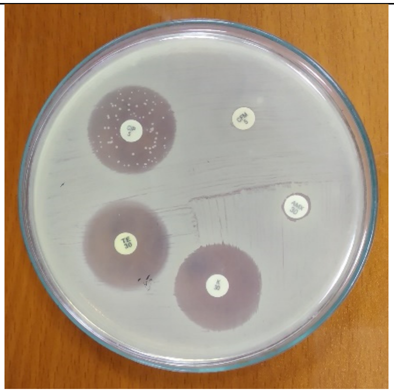</td></tr>
<tr><td colspan="2">Sample: 35</td><td colspan="2">Sample: 36</td></tr>
</table>

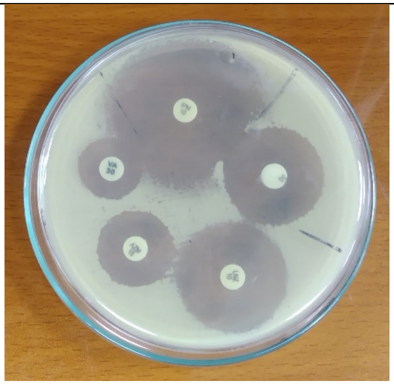	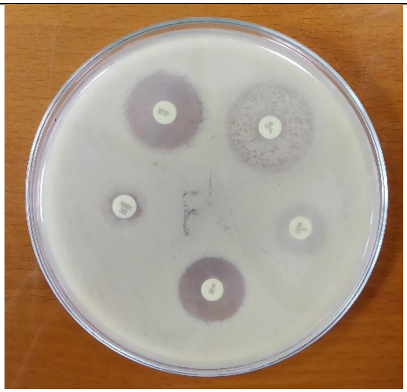	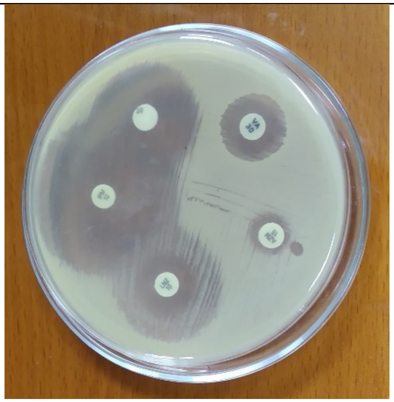	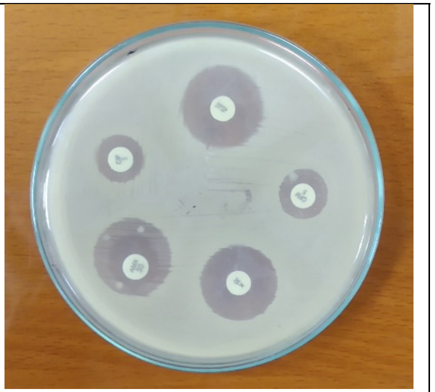
**Sample: 37**		**Sample: 38**	
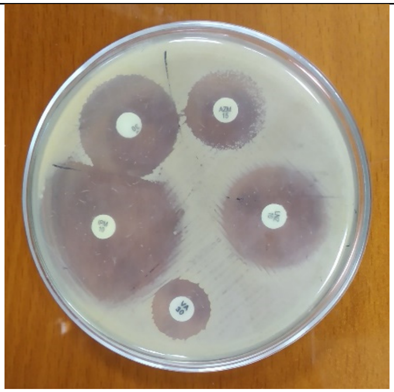	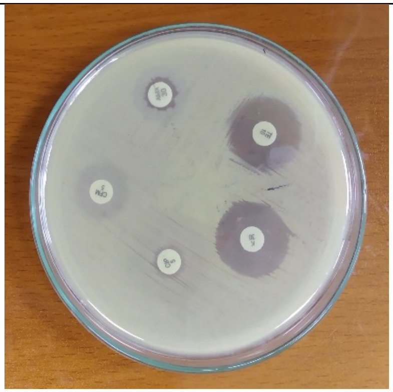	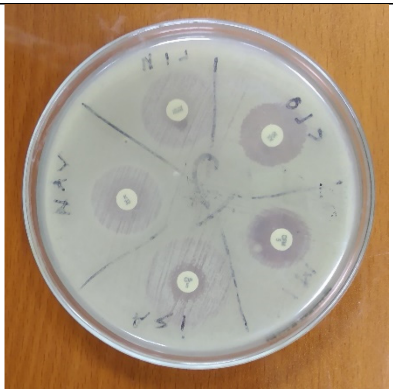	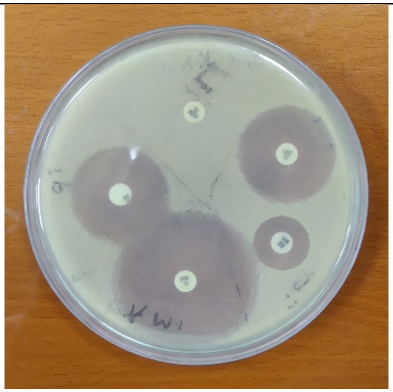
**Sample: 39**		**Sample: 40**	

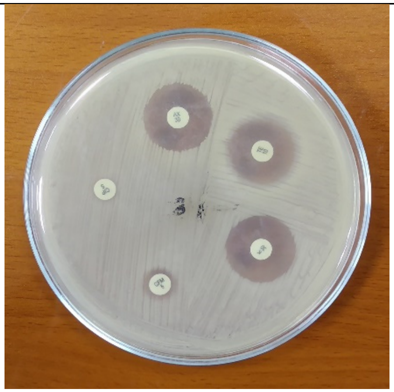
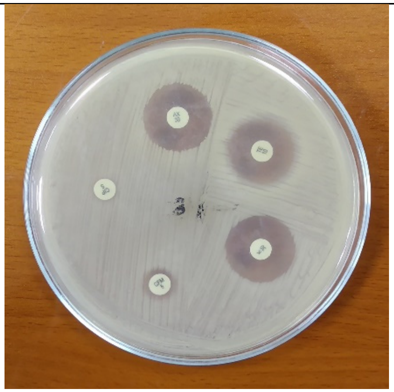
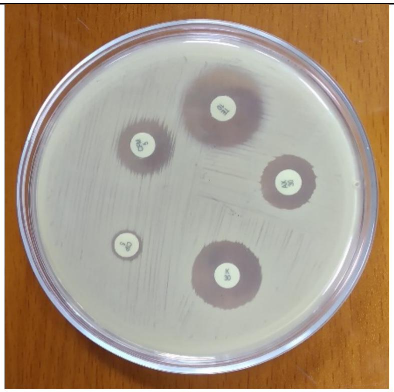
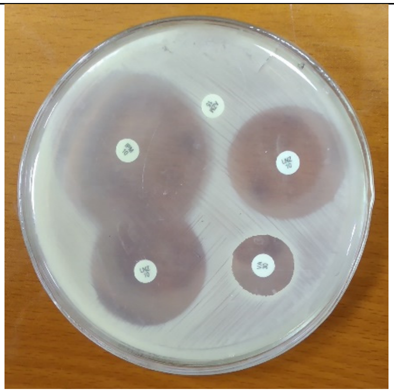

**Sample: 41**	**Sample: 42**

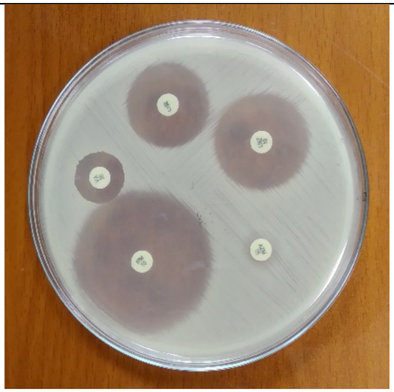
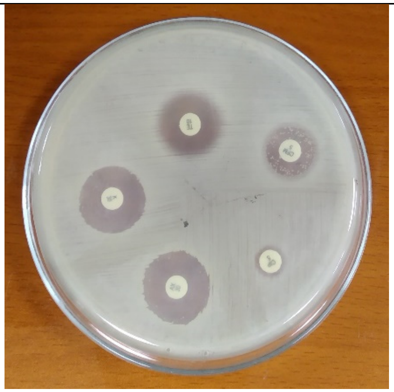
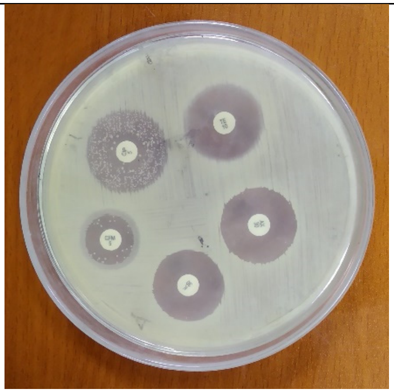
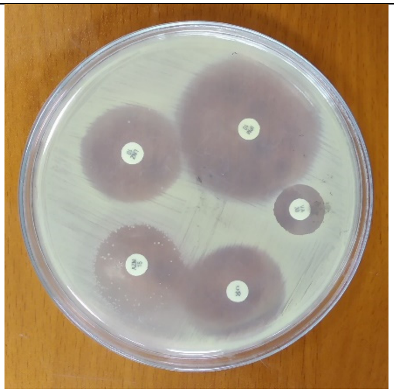

**Sample: 43**	**Sample: 44**

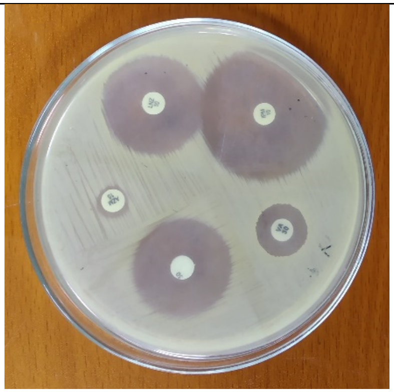	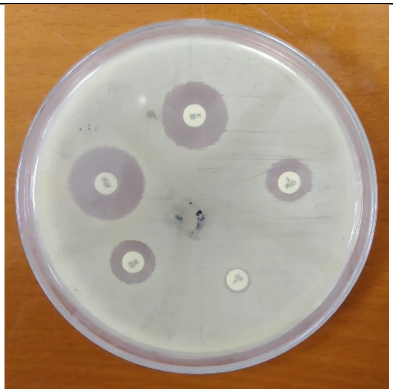	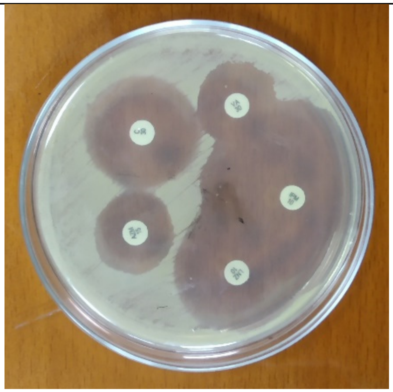	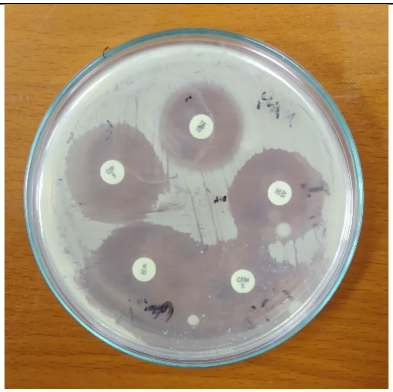
**Sample: 45**		**Sample: 46**	
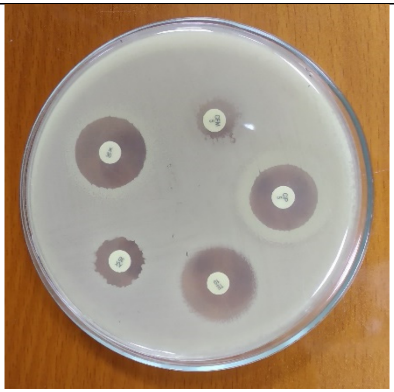	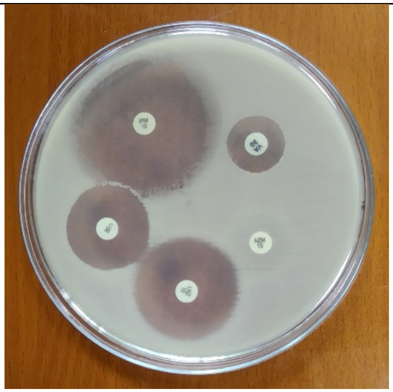	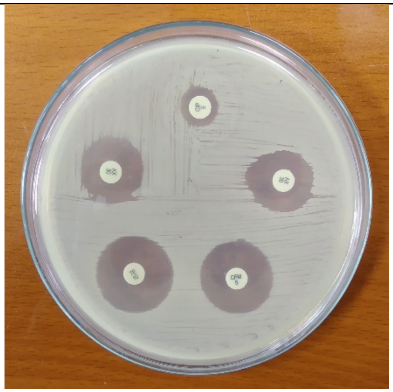	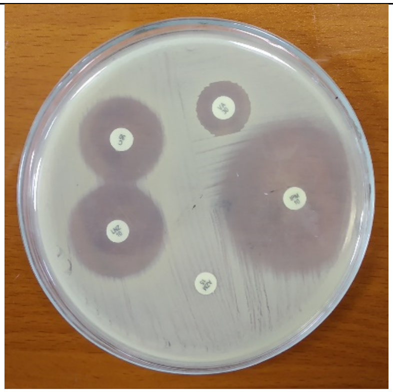
**Sample: 47**		**Sample: 48**	

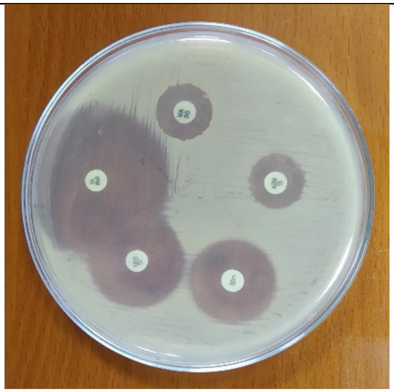
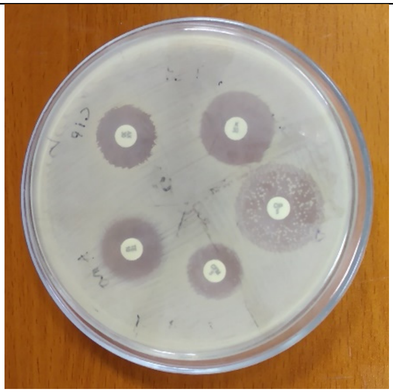

Sample: 49

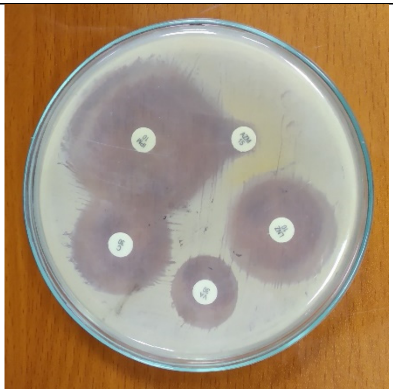
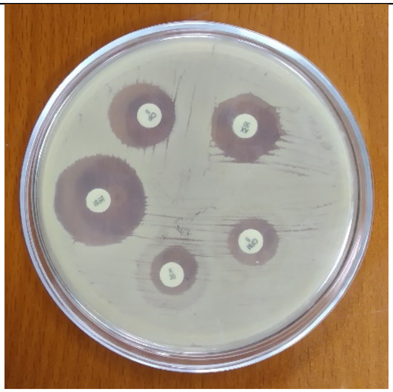

Sample: 50

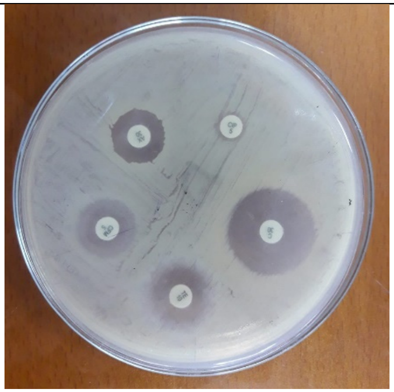
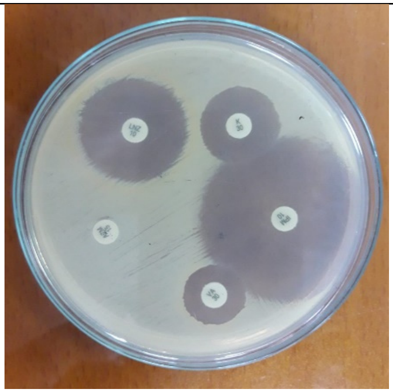

Sample: 51

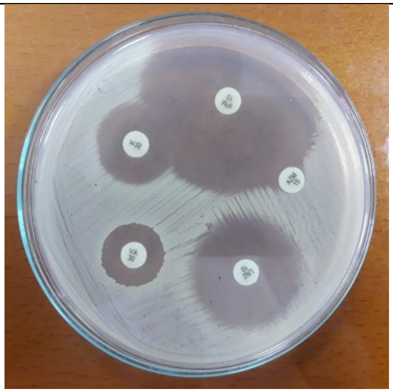
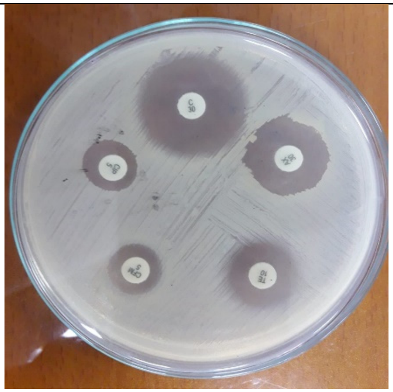

Sample: 52

<table>
<tr><td>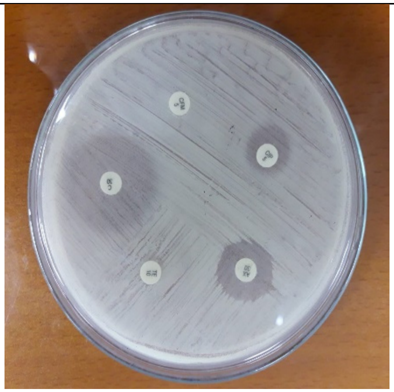</td><td>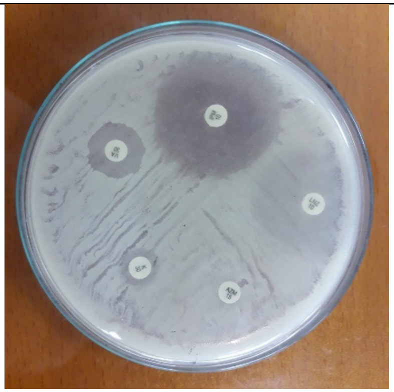</td><td>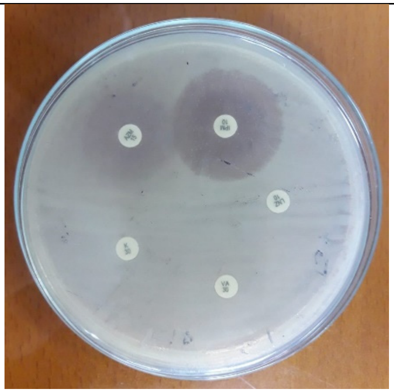</td><td>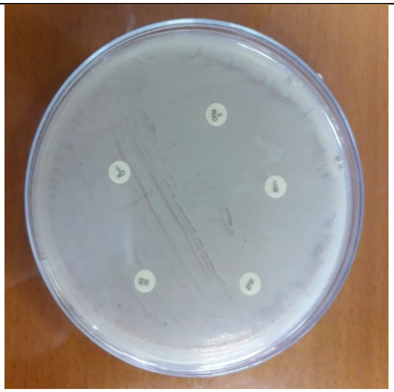</td></tr>
<tr><td colspan="2">Sample: 53</td><td colspan="2">Sample: 54</td></tr>
<tr><td>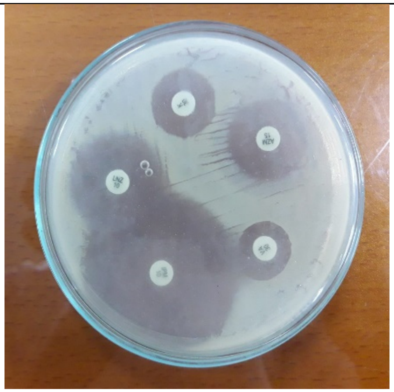</td><td>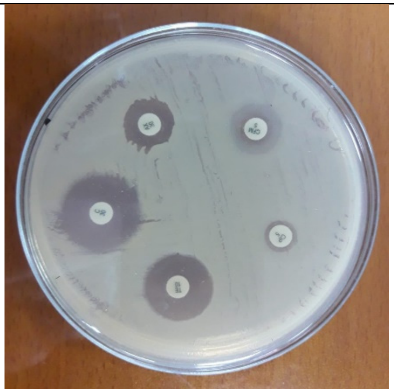</td><td>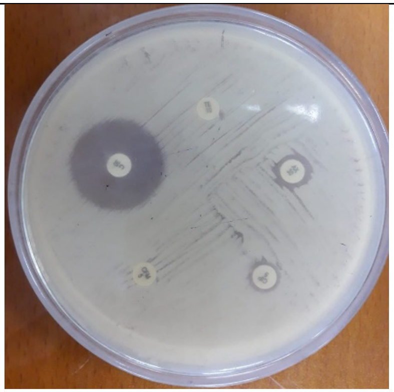</td><td>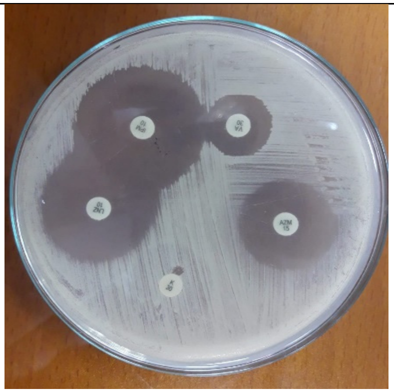</td></tr>
<tr><td colspan="2">Sample: 55</td><td colspan="2">Sample: 56</td></tr>
</table>

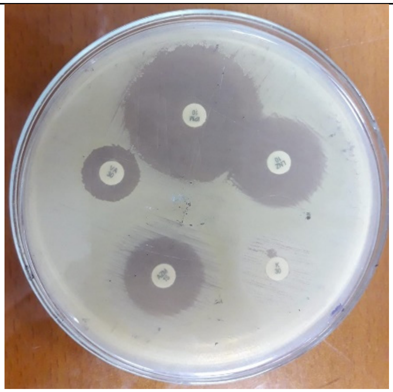
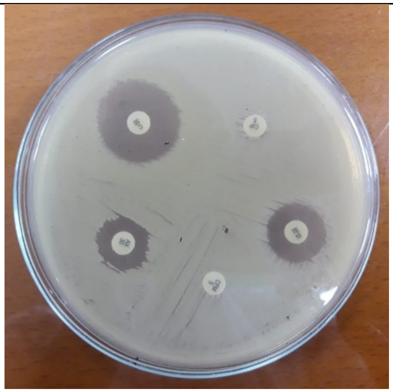

Sample: 57

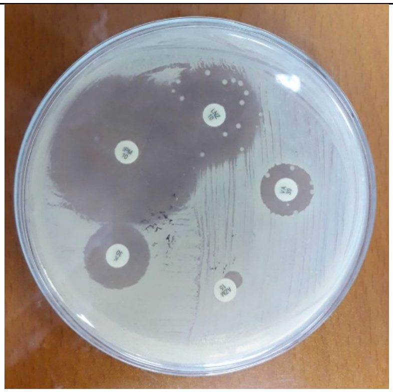
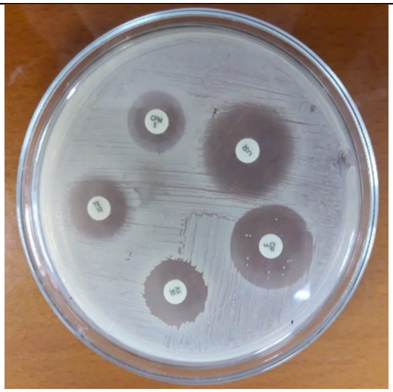

Sample: 58

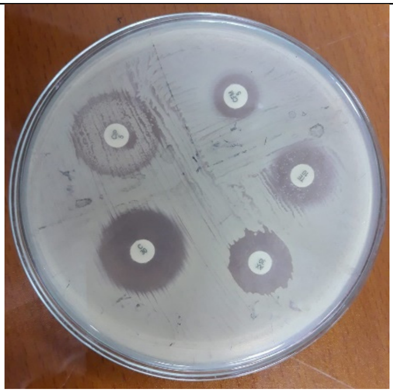
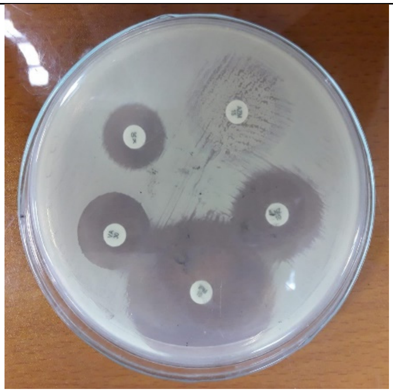

Sample: 59

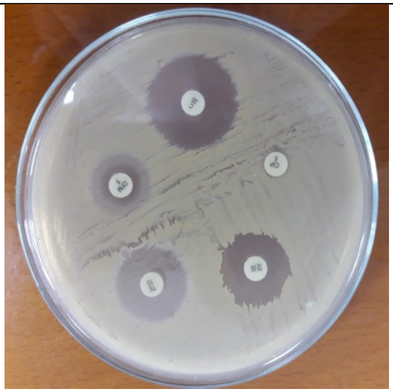
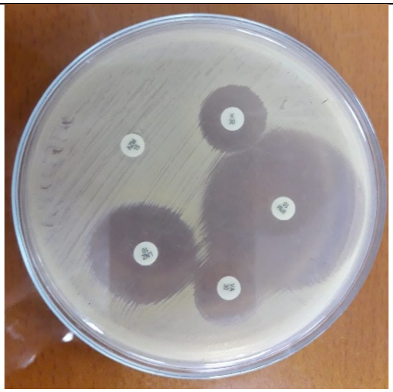

Sample: 60

Figure S1: Antibiotic Susceptibility Profile of Sixty Clinical Isolates of Staphylococcus aureus

2. **Gel doc images of all genes**

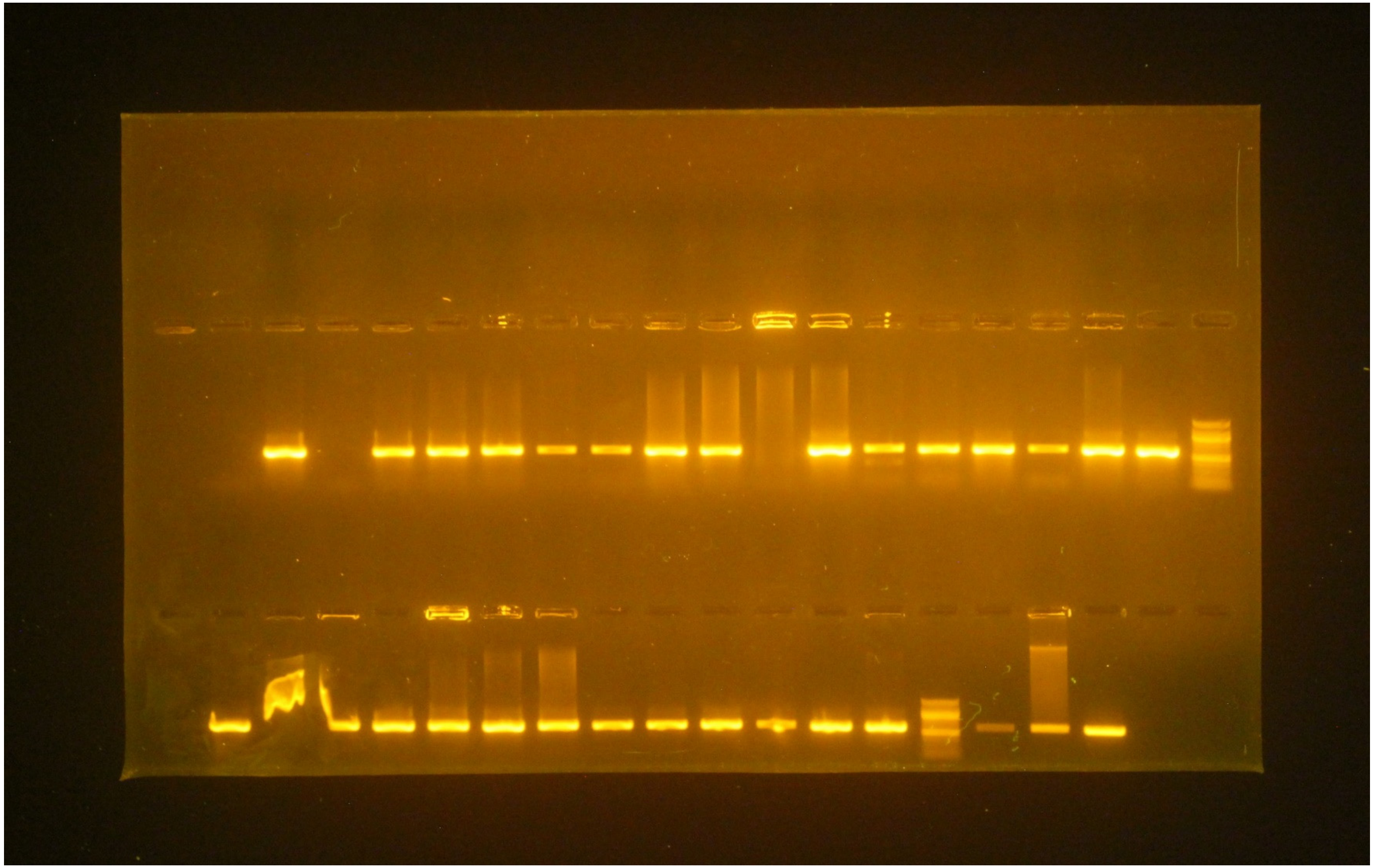

Figure S2: Detection of the Presence of norA Gene (Sample 01-30) in the Clinical Isolates of *Staphylococcus aureus*. Sample 7, 9 & 19 that were earlier checked to check the viability of the primer, were used as control in the second row.

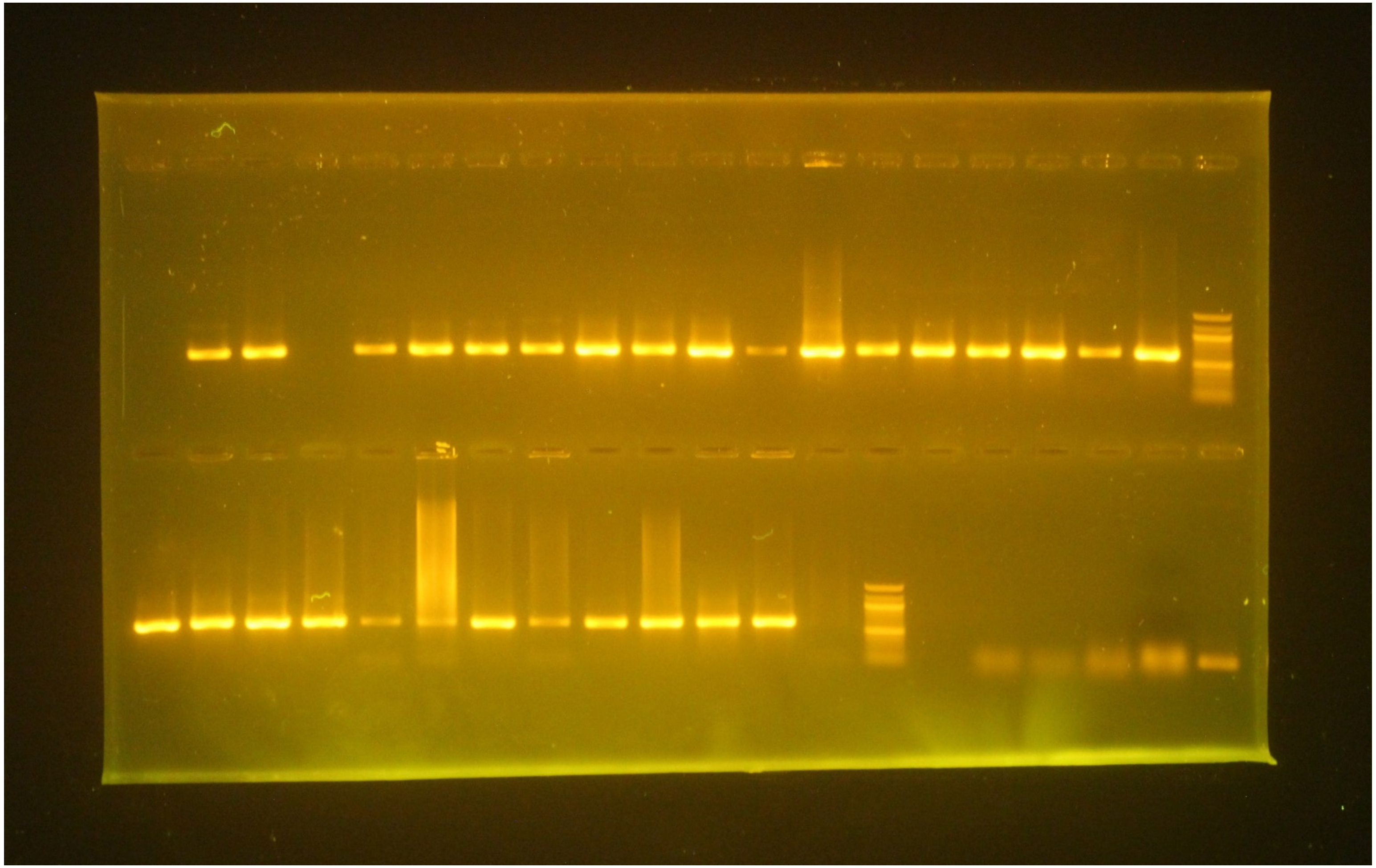

Figure S3: Detection of the Presence of norA Gene (Sample 31-60) in the Clinical Isolates of *Staphylococcus aureus*

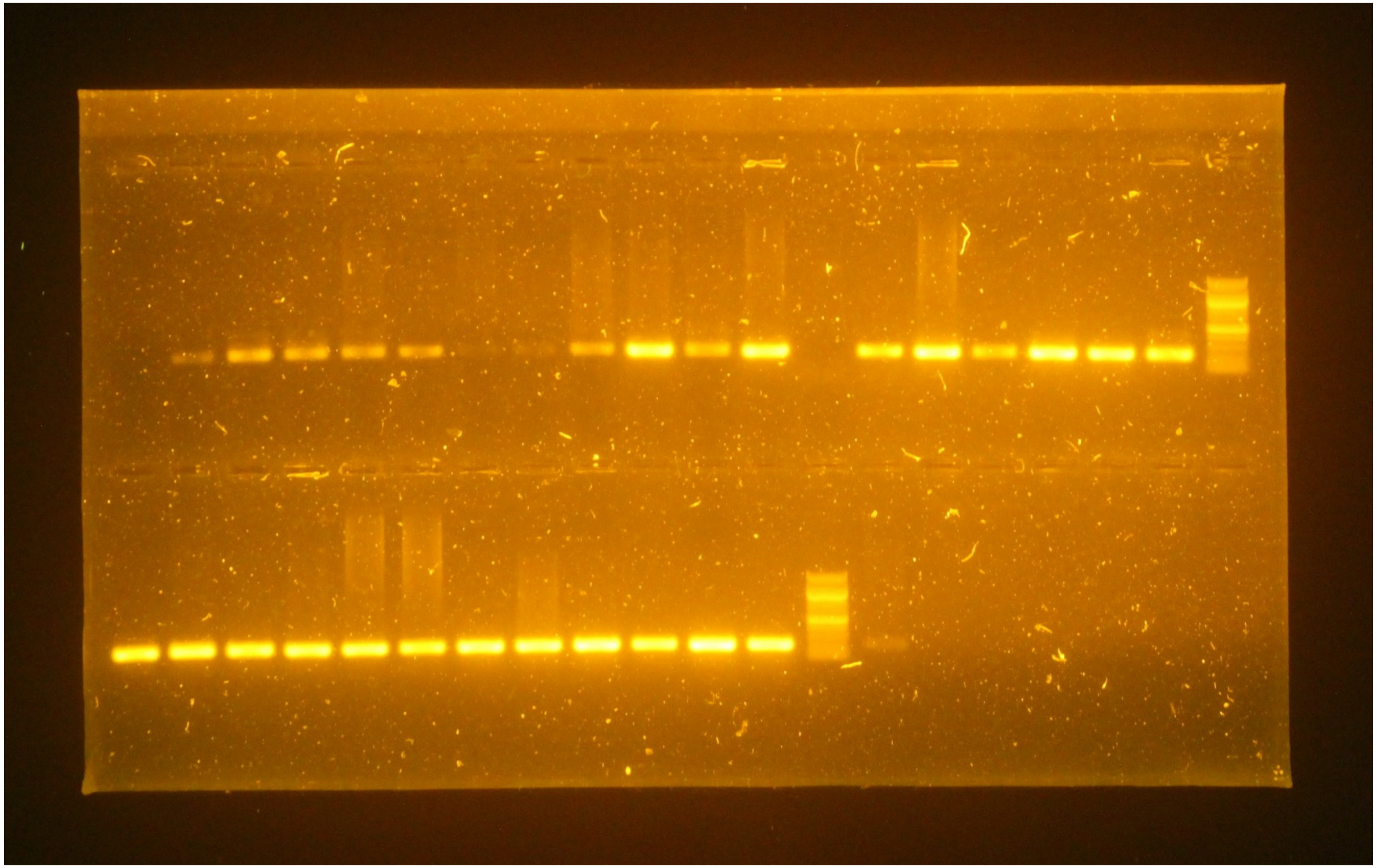

Figure S4: Detection of the Presence of norB Gene (Sample 01-30) in the Clinical Isolates of *Staphylococcus aureus*

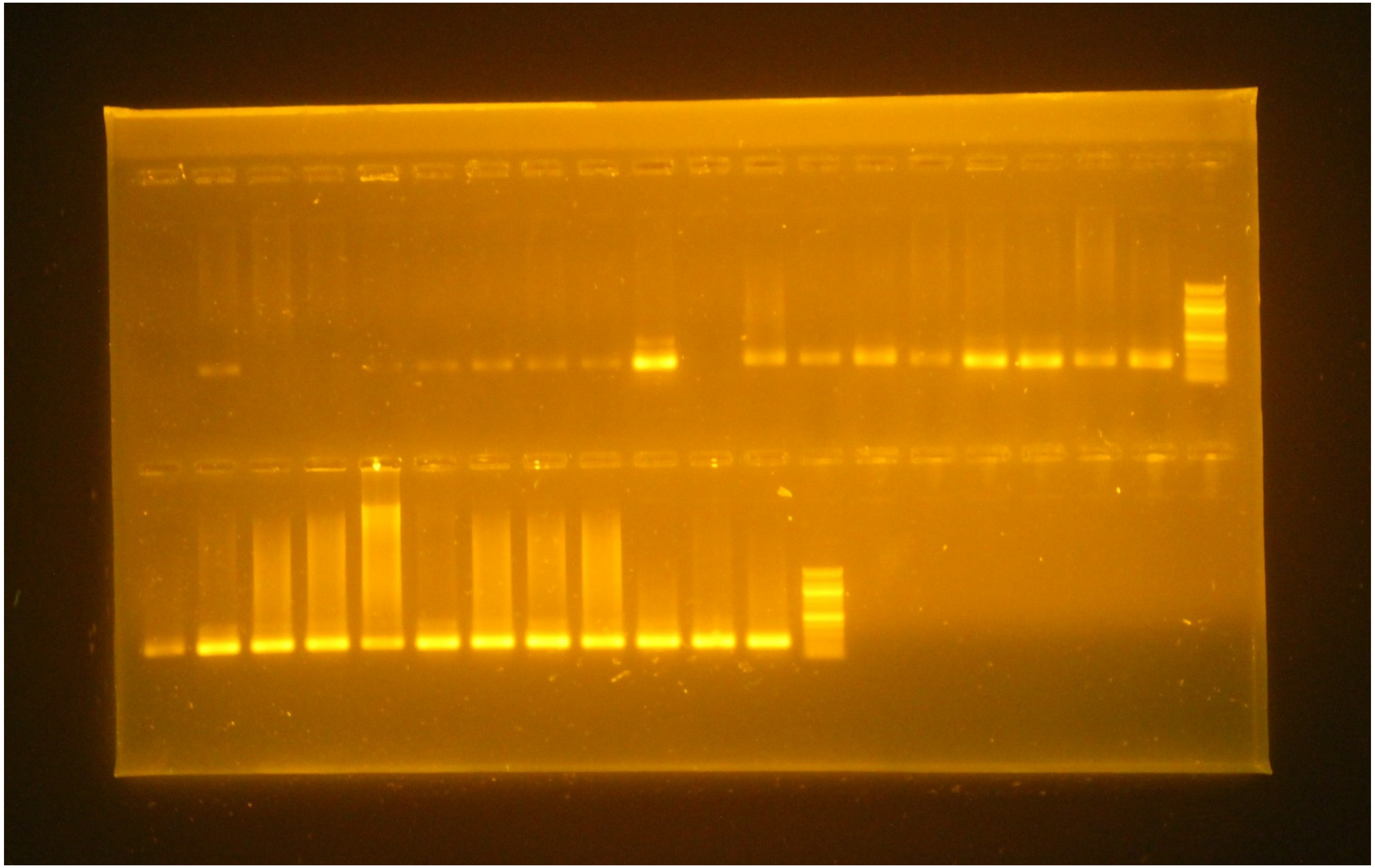

Figure S5: Detection of the Presence of norB Gene (Sample 31-60) in the Clinical Isolates of *Staphylococcus aureus*

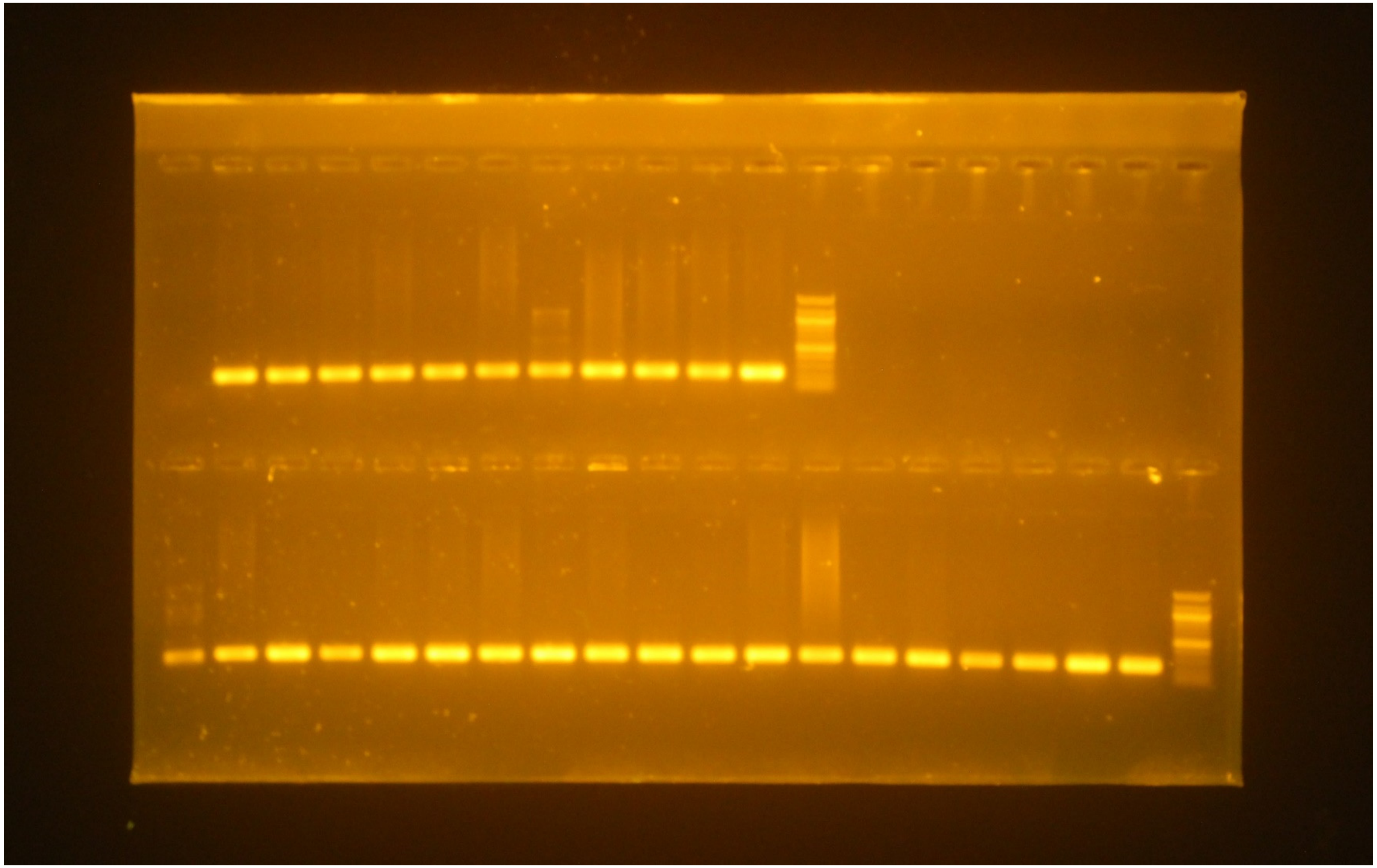

Figure S6: Detection of the Presence of norC Gene (Sample 01-30) in the Clinical Isolates of *Staphylococcus aureus*

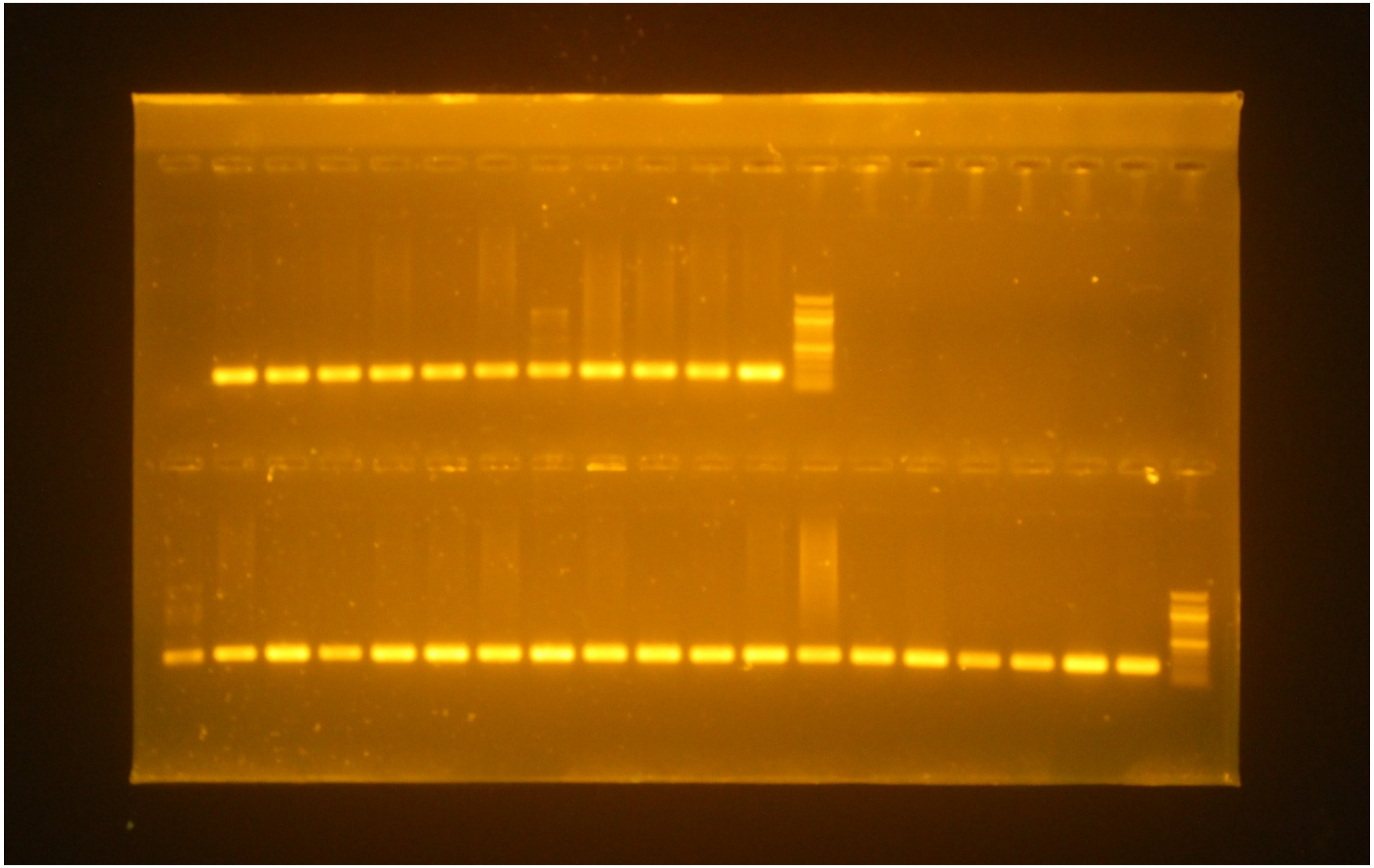

Figure S7: Detection of the Presence of norC Gene (Sample 31-60) in the Clinical Isolates of *Staphylococcus aureus*

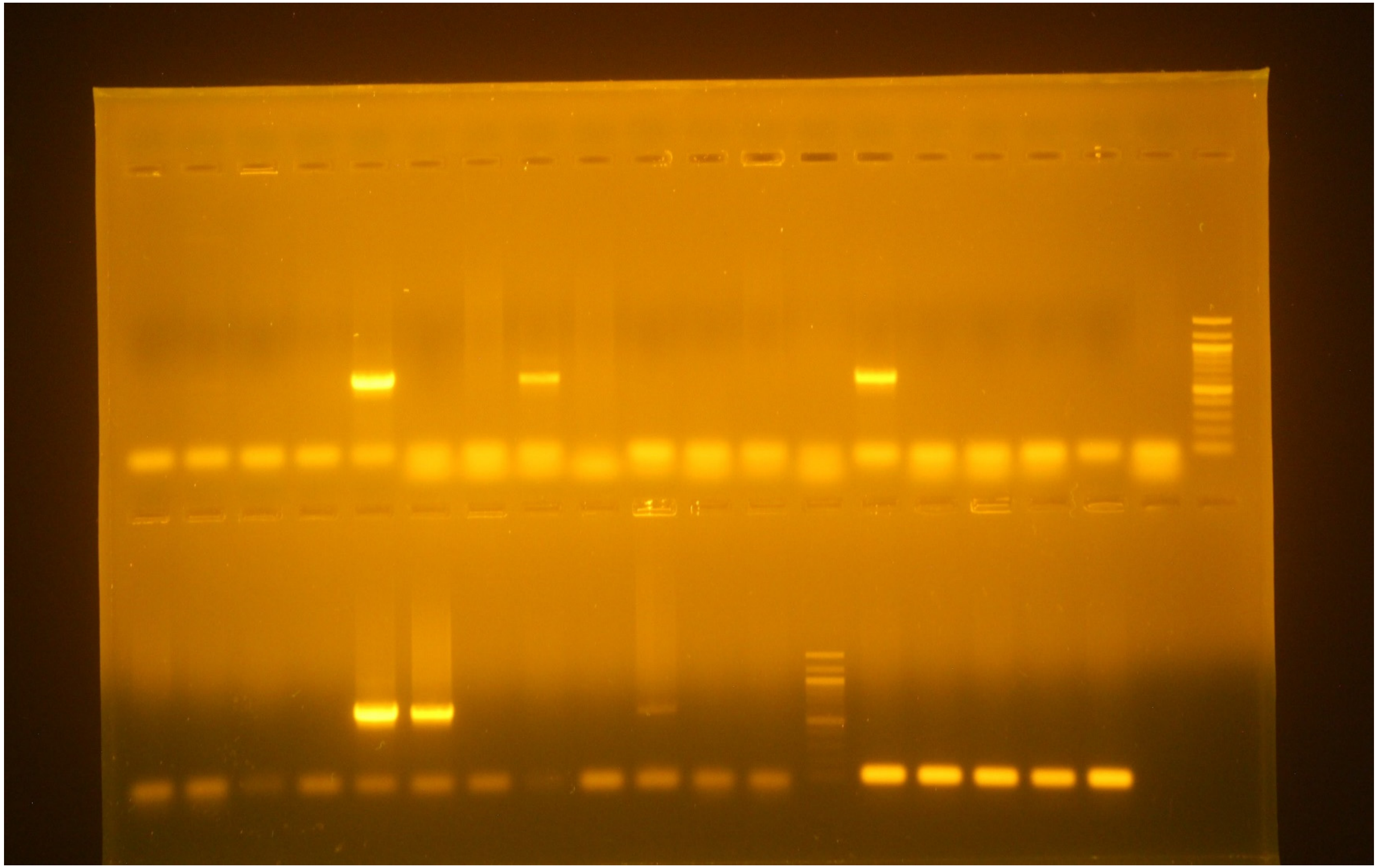

Figure S8: Detection of the Presence of qacAB Gene (Sample 1-30) and sepA (Sample 26-30) in the Clinical Isolates of *Staphylococcus aureus*

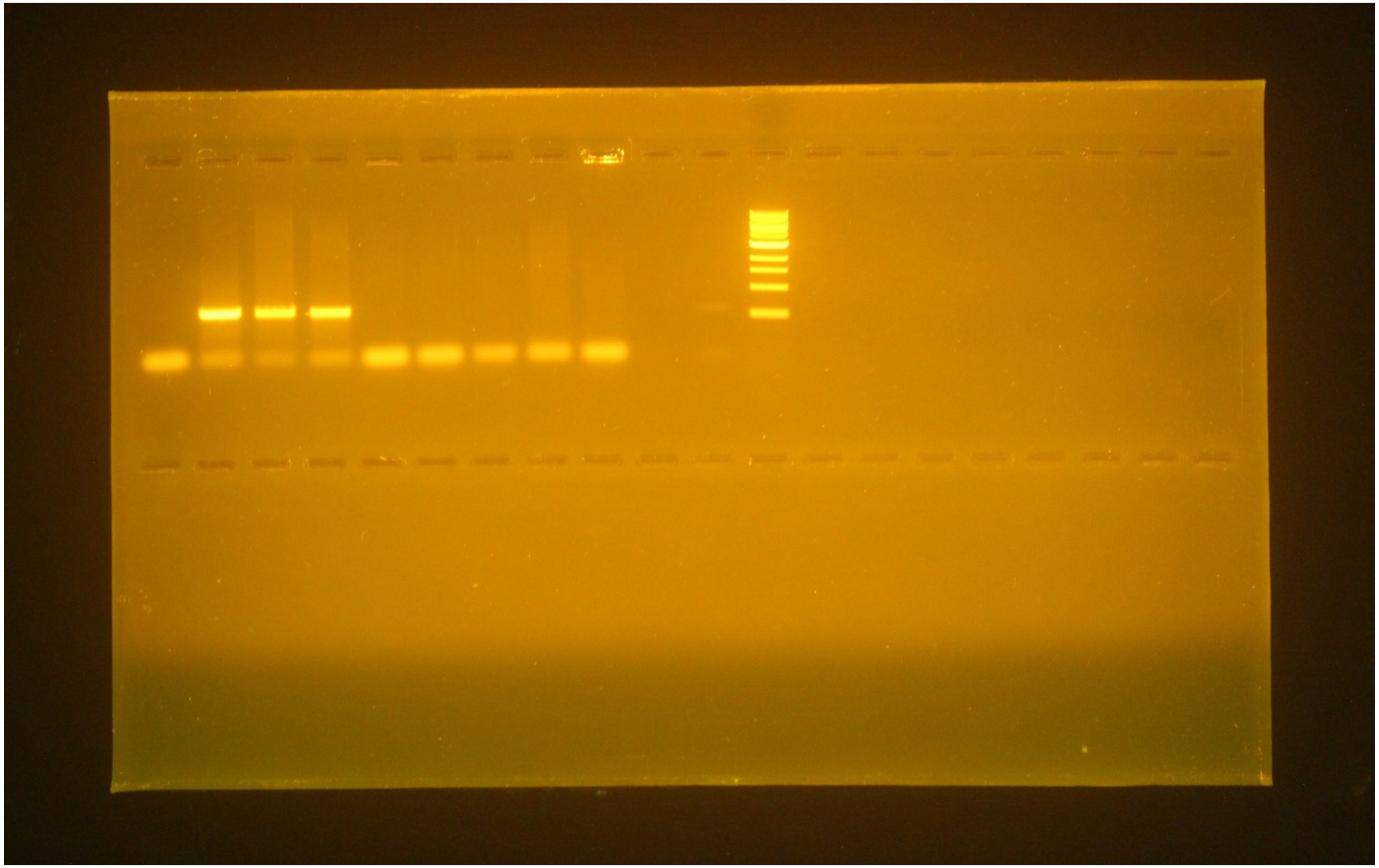

Figure S9: Detection of the Presence of qacAB Gene (Sample 31-40) in the Clinical Isolates of *Staphylococcus aureus*

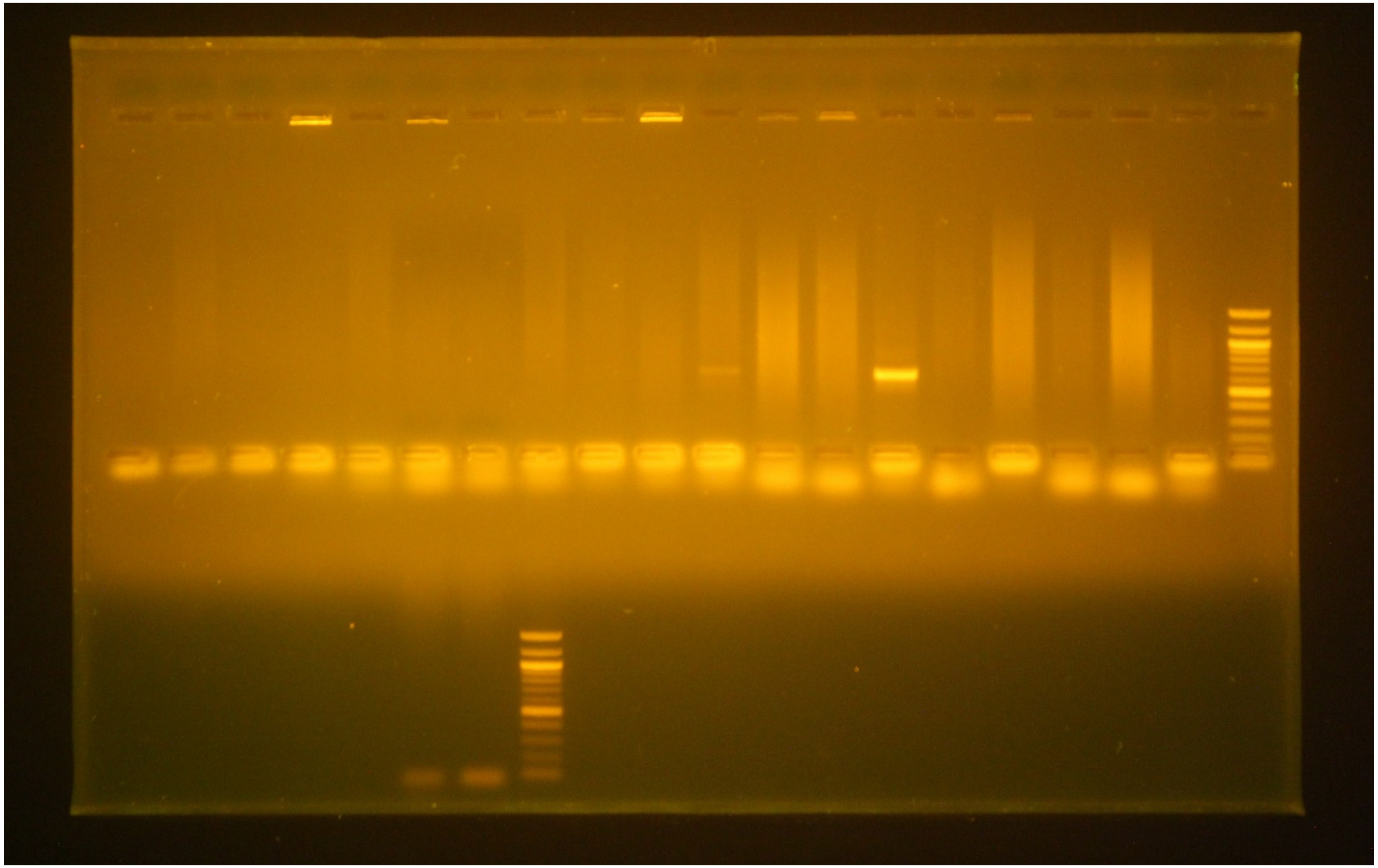

Figure S10: Detection of the Presence of qacAB Gene (Sample 41-60) in the Clinical Isolates of *Staphylococcus aureus*

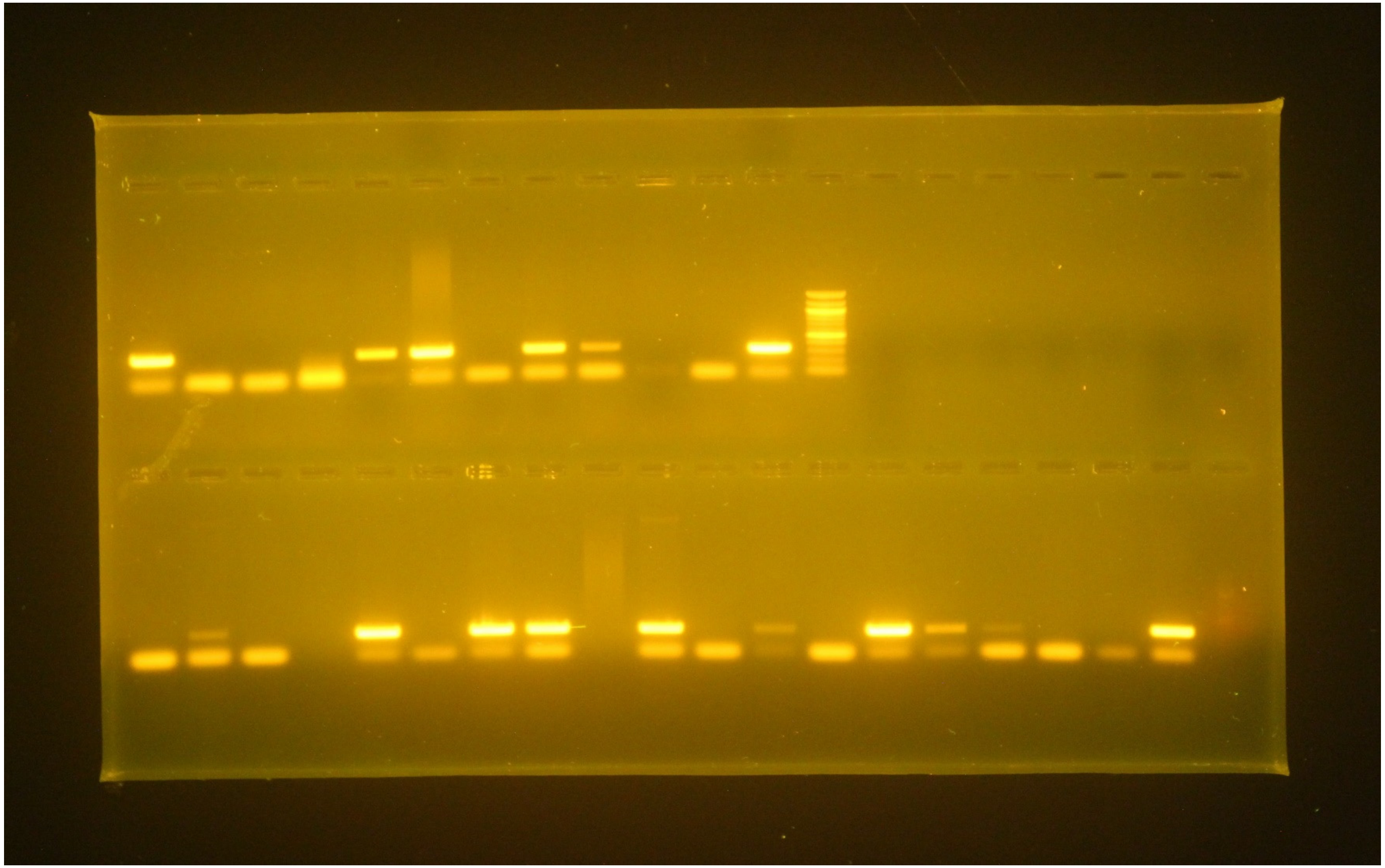

Figure S11: Detection of the Presence of mecA Gene (Sample 1-30) in the Clinical Isolates of *Staphylococcus aureus*

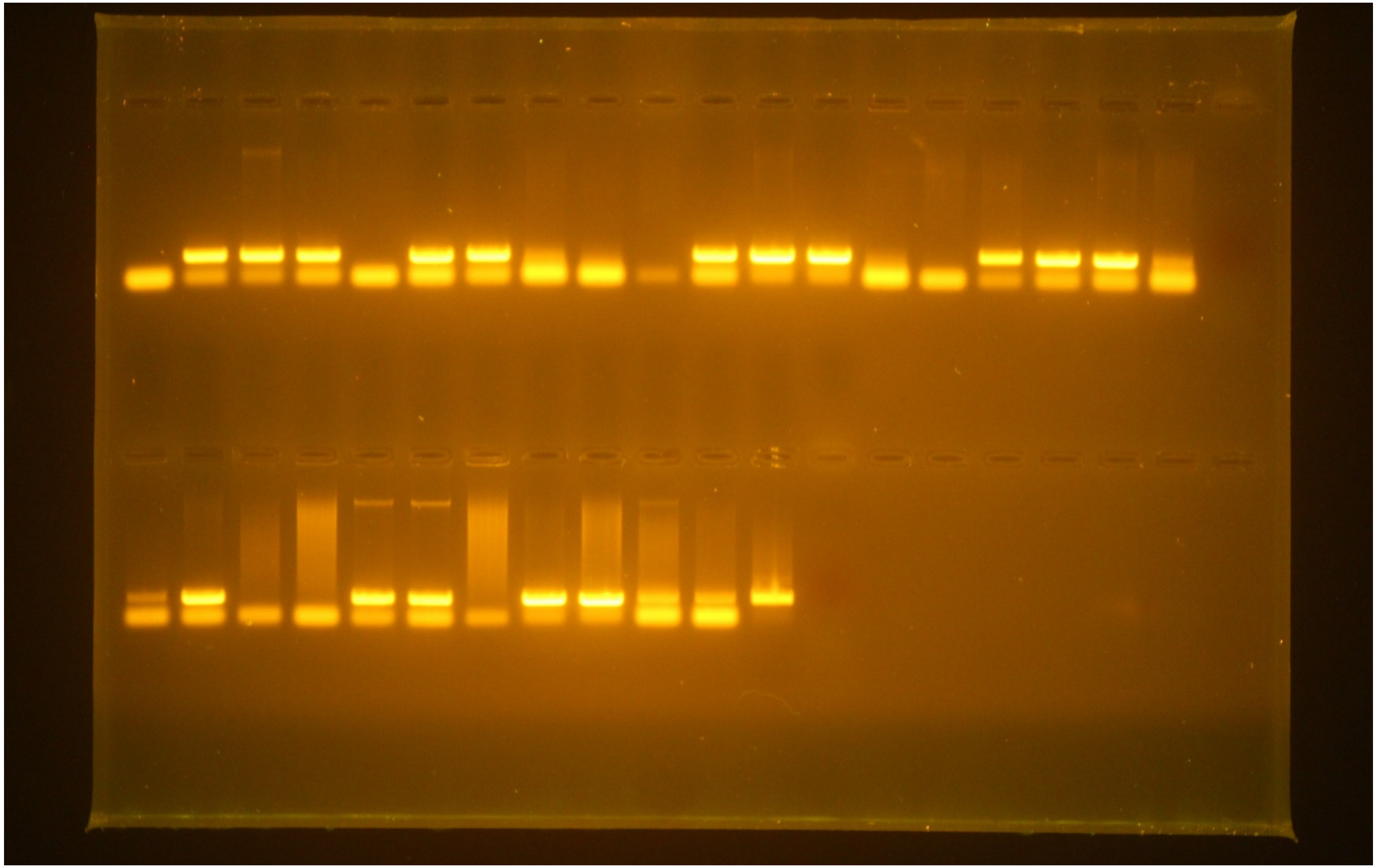

Figure S12: Detection of the Presence of mecA Gene (Sample 31-60) in the Clinical Isolates of *Staphylococcus aureus*

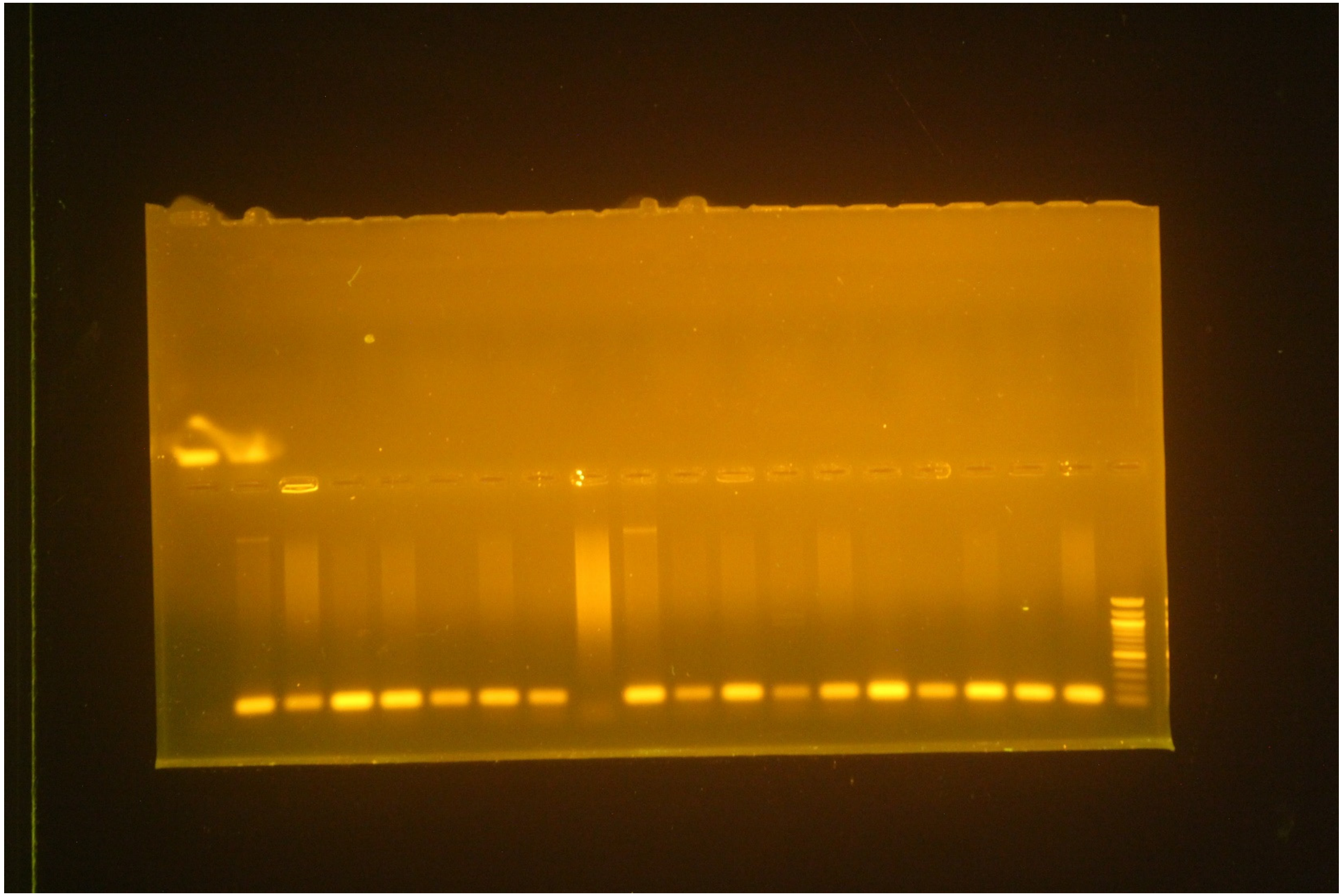

Figure S13: Detection of the Presence of sepA Gene (Sample 1-18) in the Clinical Isolates of *Staphylococcus aureus*

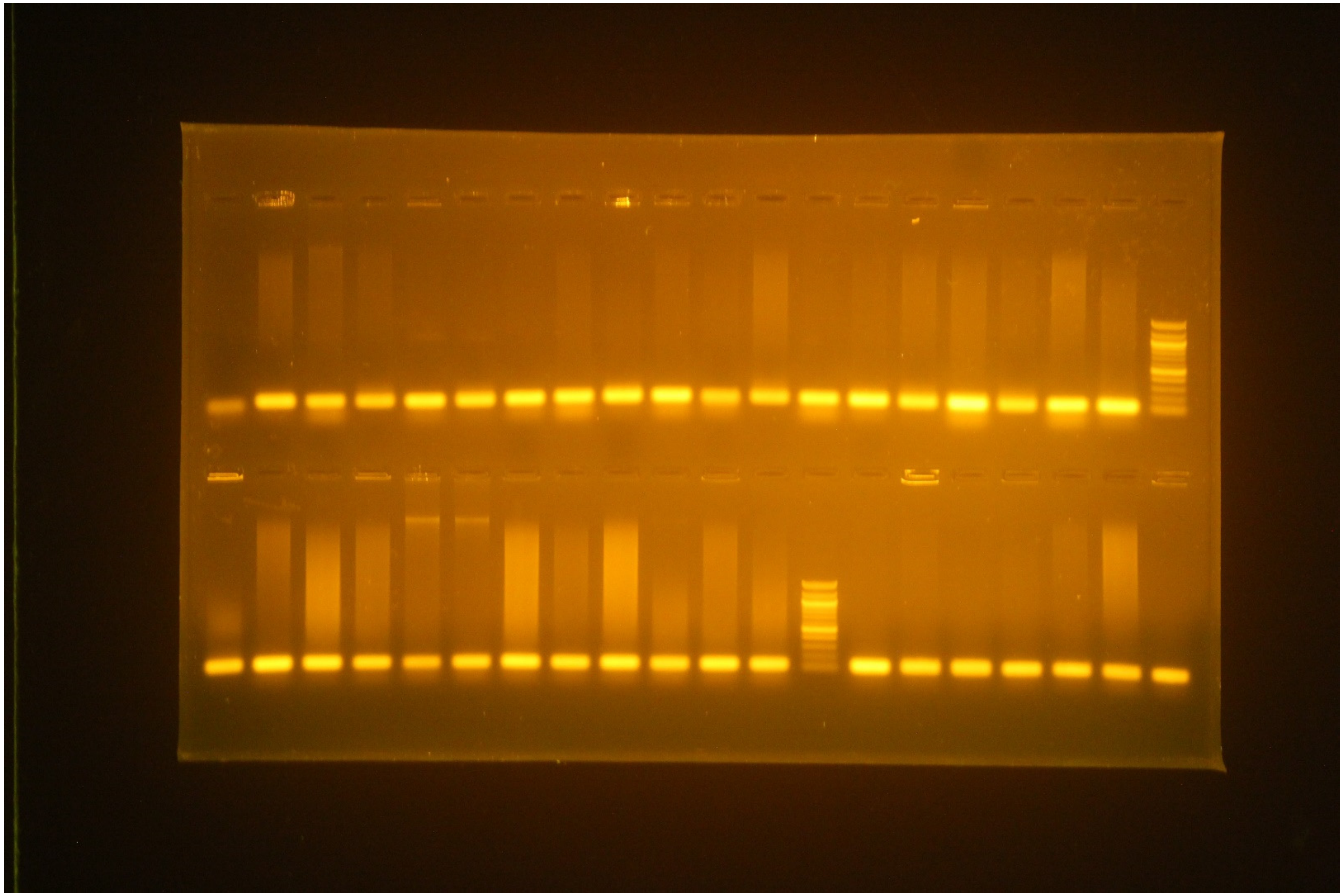

Figure S14: Detection of the Presence of sepA Gene (Sample 31-60; 19-25) in the Clinical Isolates of *Staphylococcus aureus*

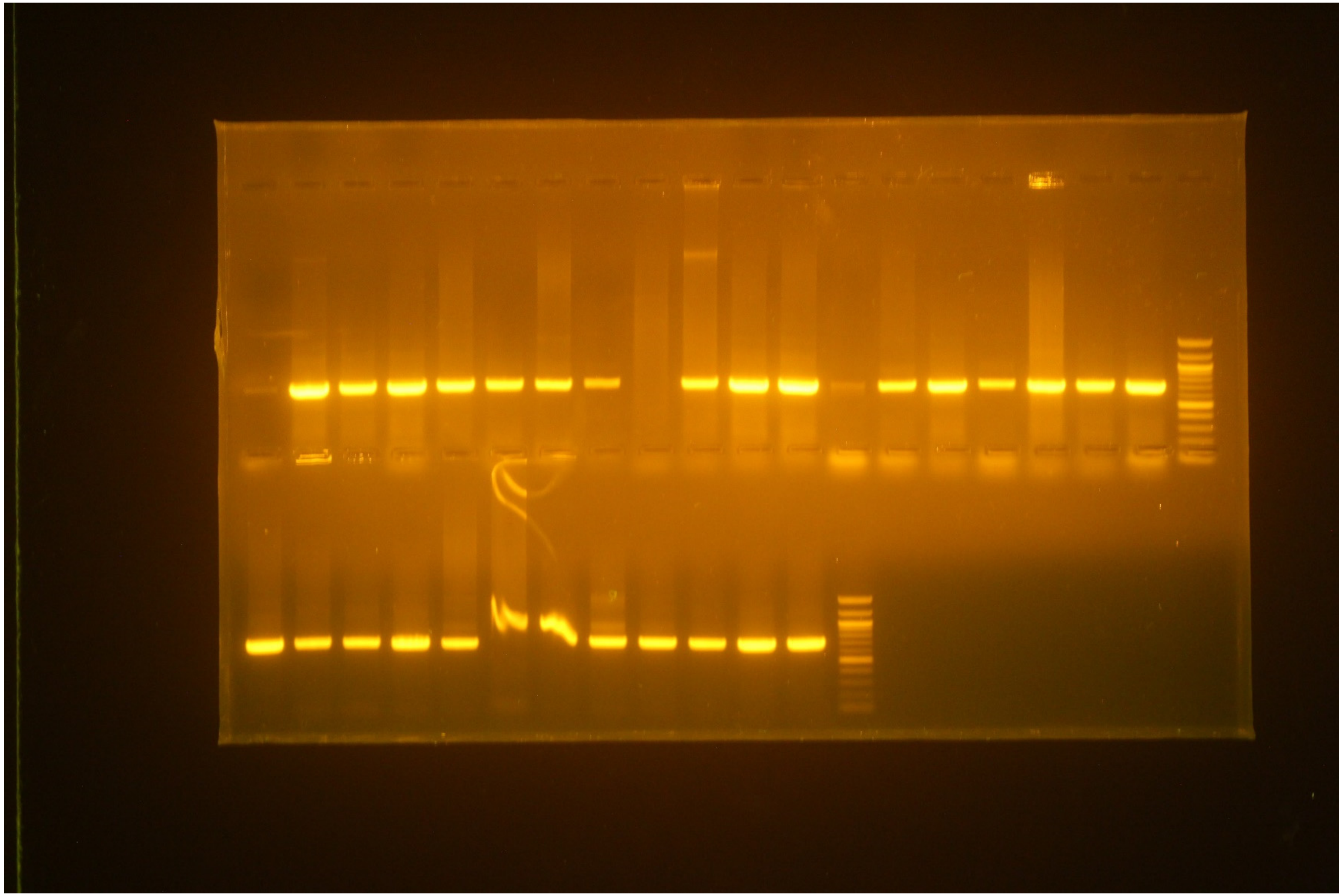

Figure S15: Detection of the Presence of mepA Gene (Sample 1-30) in the Clinical Isolates of *Staphylococcus aureus*

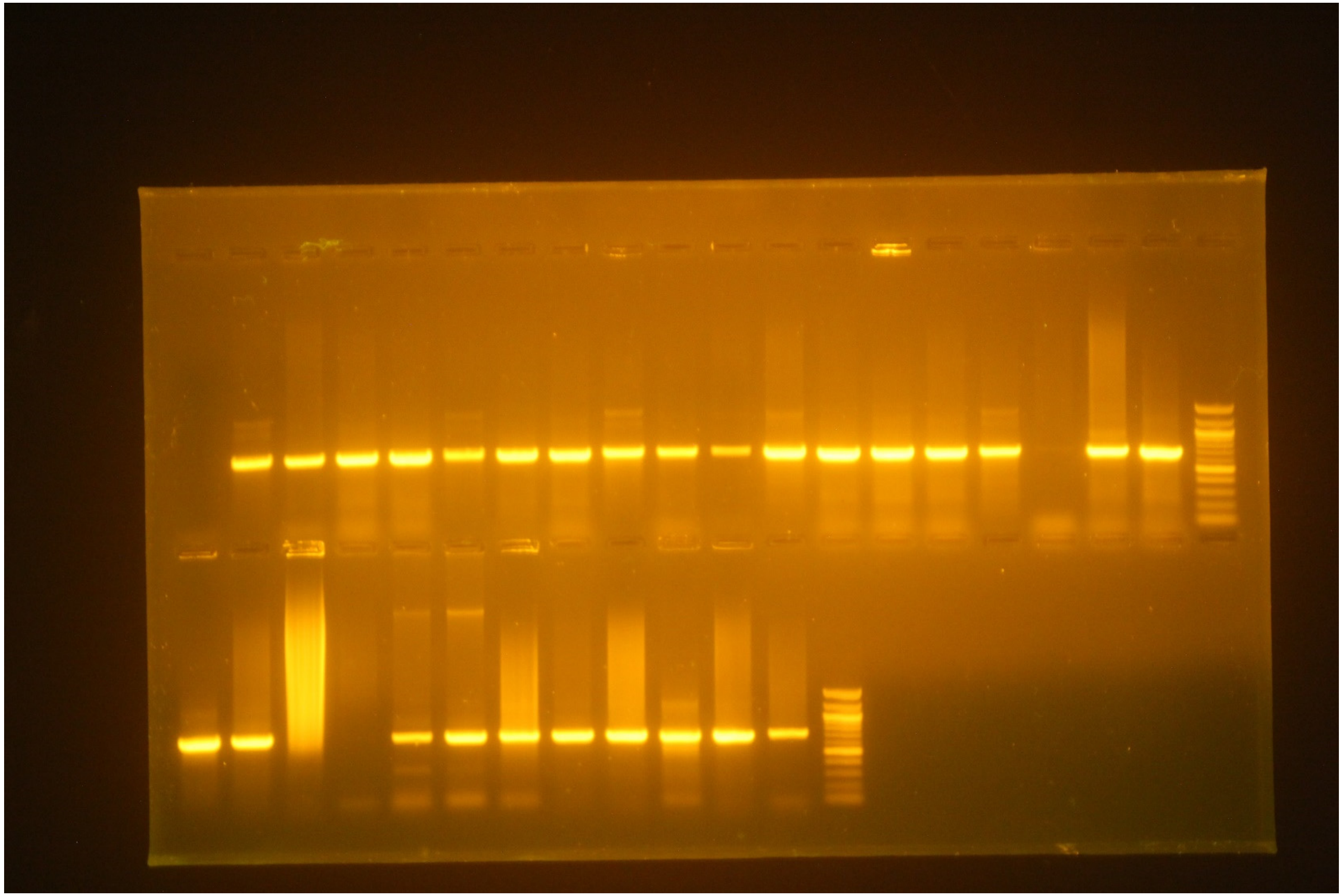

Figure S16: Detection of the Presence of mepA Gene (Sample 31-60) in the Clinical Isolates of *Staphylococcus aureus*

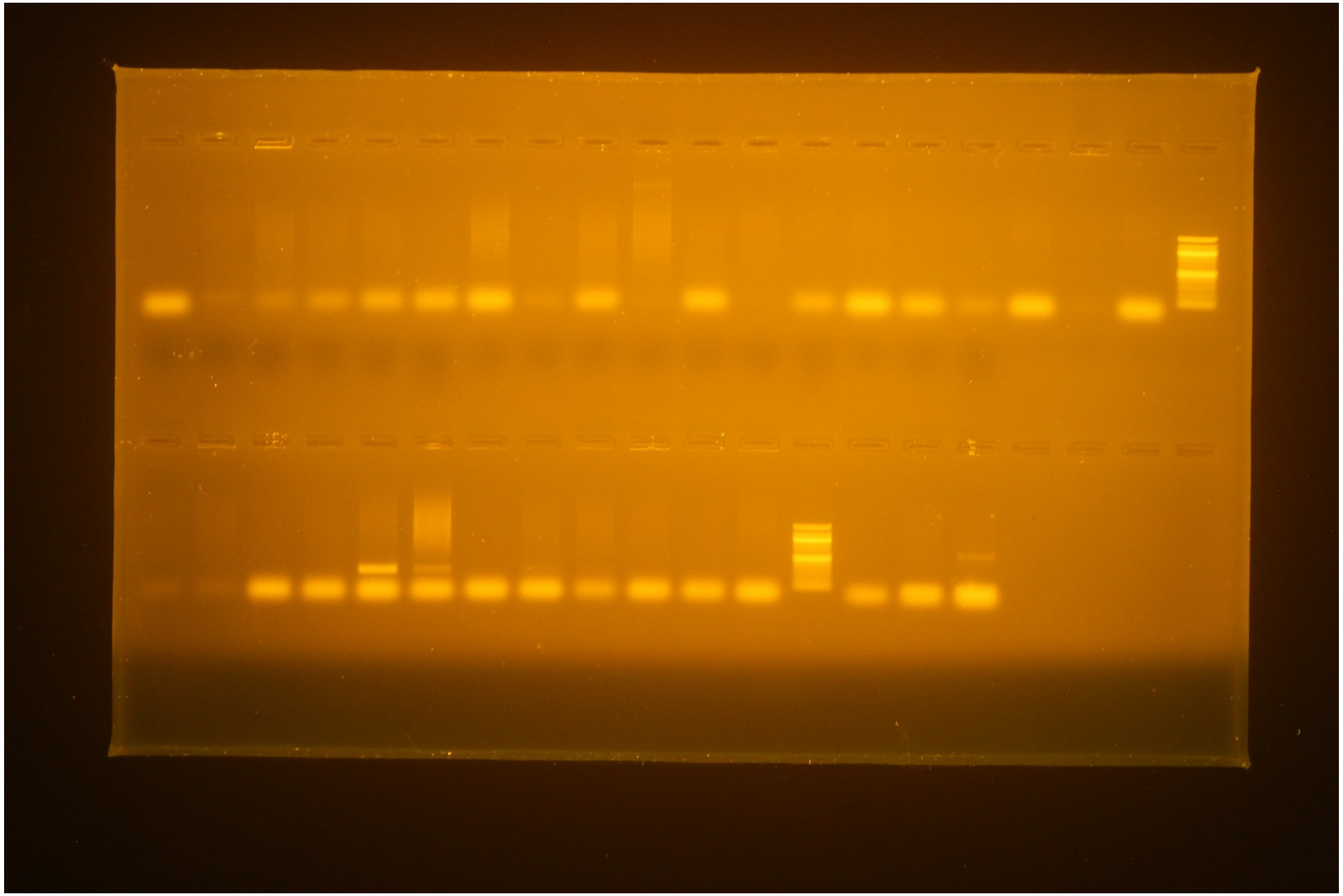

Figure S17: Detection of the Presence of smr Gene (Sample 1-30) in the Clinical Isolates of *Staphylococcus aureus*

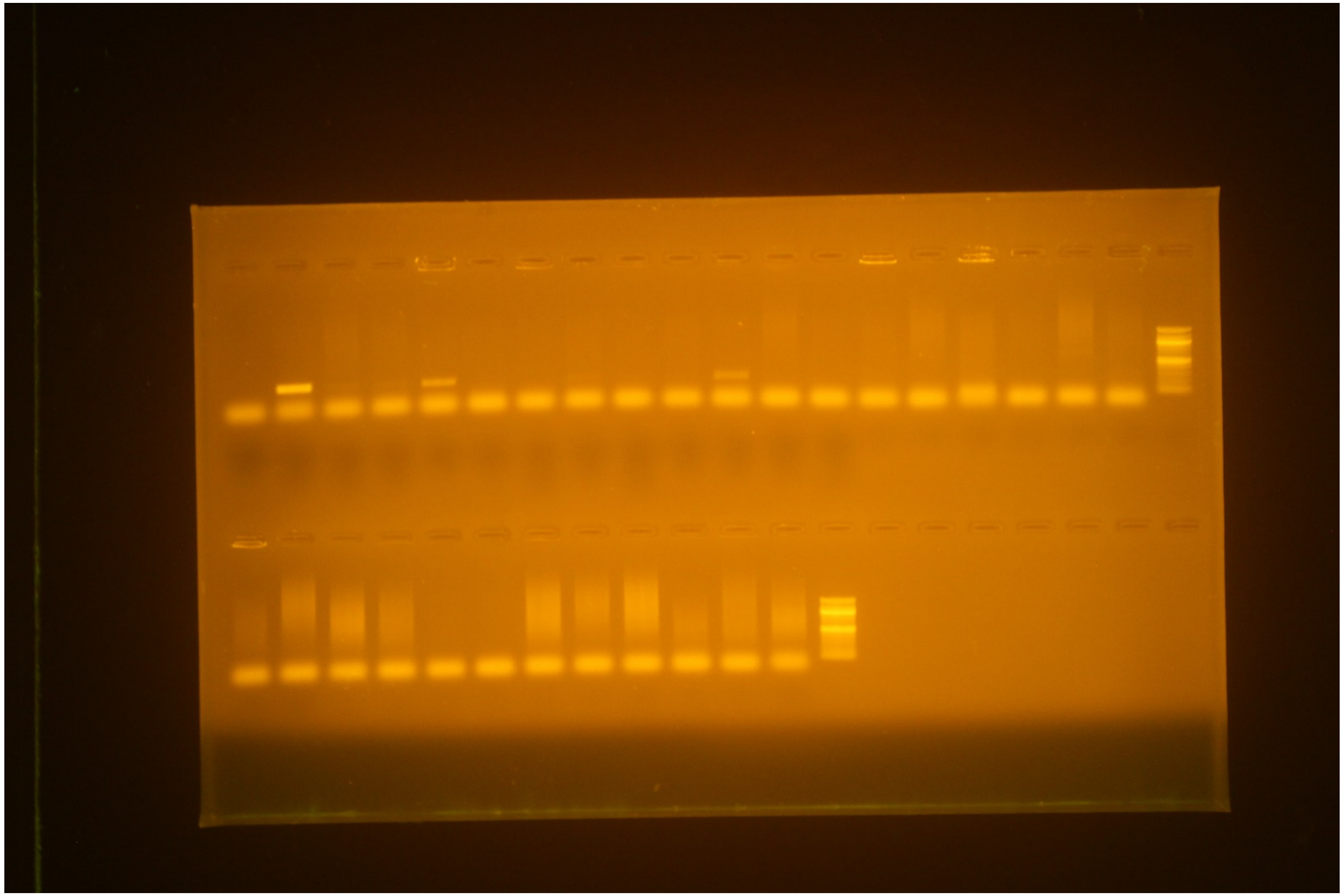

Figure S18: Detection of the Presence of smr Gene (Sample 31-60) in the Clinical Isolates of *Staphylococcus aureus*

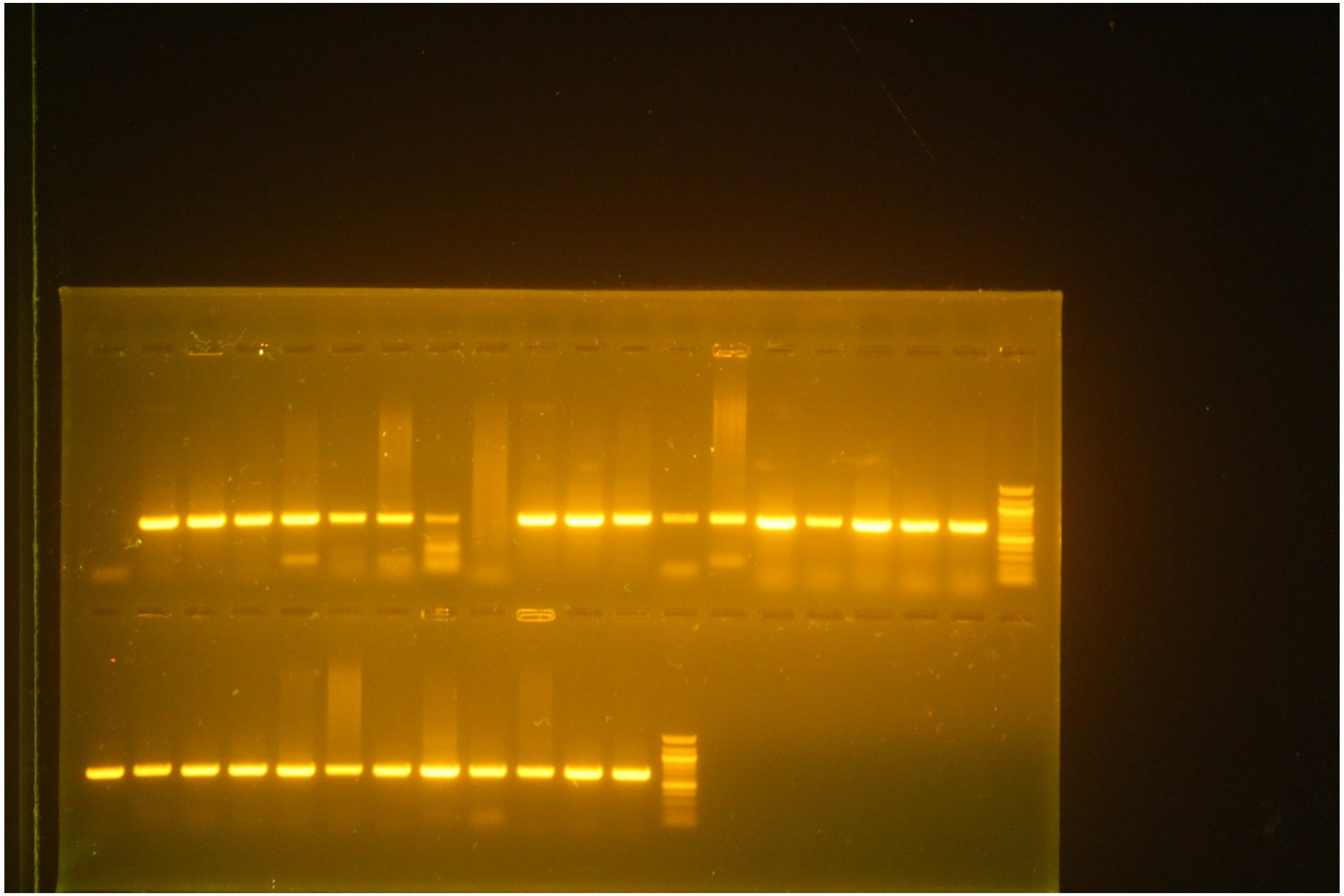

Figure S19: Detection of the Presence of mdeA Gene (Sample 1-30) in the Clinical Isolates of *Staphylococcus aureus*

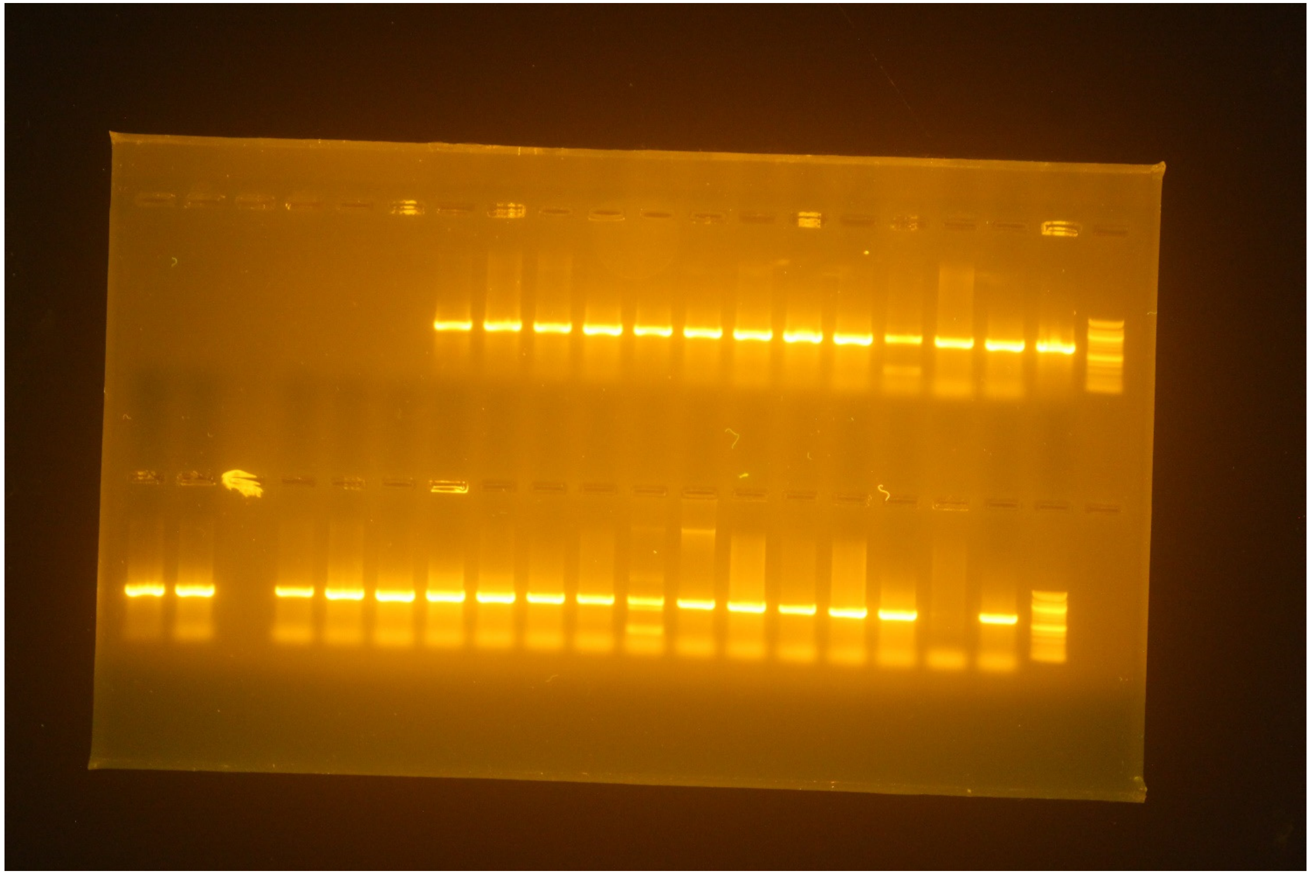

Figure S20: Detection of the Presence of mdeA Gene (Sample 31-60) in the Clinical Isolates of *Staphylococcus aureus*
